# Transcriptome profiles reveal that gibberellin-related genes regulate weeping traits in crape myrtle

**DOI:** 10.1038/s41438-020-0279-3

**Published:** 2020-04-01

**Authors:** Suzhen Li, Tangchun Zheng, Xiaokang Zhuo, Zhuojiao Li, Lulu Li, Ping Li, Like Qiu, Huitang Pan, Jia Wang, Tangren Cheng, Qixiang Zhang

**Affiliations:** 1 Beijing Advanced Innovation Center for Tree Breeding by Molecular Design, Beijing Forestry University, Beijing, 100083 China; 2 Beijing Key Laboratory of Ornamental Plants Germplasm Innovation and Molecular Breeding, National Engineering Research Center for Floriculture, Beijing Laboratory of Urban and Rural Ecological Environment, Engineering Research Center of Landscape Environment of the Ministry of Education, Key Laboratory of Genetics and Breeding in Forest Trees and Ornamental Plants of Ministry of Education, School of Landscape Architecture, Beijing Forestry University, Beijing, 100083 China

**Keywords:** Molecular engineering in plants, Plant molecular biology

## Abstract

Plant architecture includes vital traits that influence and benefit crops, and economically important trees. Different plant architectures provide natural beauty. Weeping ornamental plants are aesthetically appealing to people. The regulatory mechanism controlling the weeping trait is poorly understood in crape myrtle. To investigate the weeping trait mechanism, transcriptional profiling of different organs in weeping and upright crape myrtle was performed based on phenotype. Phenotypic and histological analyses demonstrated that endodermal cells were absent, and that new shoot phenotypes could be rescued by the GA3 treatment of weeping plants. The transcriptional analysis and coexpression network analysis (WGCNA) of differentially expressed genes indicated that GA synthesis and signal transduction pathways play a role in weeping traits. When the expression level of a negative element of GA signaling, *LfiGRAS1*, was reduced by virus-induced gene silencing (VIGS), new branches grew in infected plants in a negatively geotropic manner. An integrated analysis implied that GA had a strong influence on weeping crape myrtle by interacting with other factors. This study helps to elucidate the mechanism governing the weeping trait and can improve the efficiency of breeding in *Lagerstroemia*.

## Introduction

Plant architecture is a vital characteristic that can contribute to economically valuable properties of crops and orchards. Among ornamental plants, varied plant architectures provide natural beauty^1^. Controlling plant architecture is considered to be a significant objective in plant breeding^2^. As a result, considerable progress in plant architecture research has been made in the past several years. Weeping branches are one of the most conspicuous plant architecture characteristics and have attracted increasing attention. However, the molecular mechanism governing the weeping trait has not been elucidated. Therefore, studying the molecular basis of the weeping trait is a central goal in breeding.

Many environmental signals, including light and gravity, can influence plant architecture, but genes also play an important role in plant architecture^2,3^. *LAZY1* has been identified in many plants and can stimulate the upward growth of plants through gravitropic response pathways. In rice, *Arabidopsis*, and maize, *LAZY1* mutations result in wide shoot angles^4–8^. In poplar, the overexpression of the *LAZY1* gene reduces branch angles, whereas the overexpression of *tiller angle control 1* (*TAC1*) increased the branch angle and promoted an outward branch growth orientation^9^. Similarly, plants with reduced or no *TAC1* expression exhibit more vertical shoot angles, as observed in *Arabidopsis*, maize, rice, and peach^10–14^. Hollender et al.^15^ identified a *WEEP* gene for weeping peach and suggested that it might play a major role in controlling directional growth. Another major gene, *loose plant architecture 1* (*LAP1*), affects the leaf petiole angle in soybean. In rice, *LAP1* contributes to gravitropic responses and regulates both tiller and leaf angles^16,17^. Rice mutants in which the *rostrate growth 1* (*PROG1*) gene is disrupted show more upright growth^18,19^. The rice *phytochrome-interacting factor* (*PIF*) *OSPIL15* plays a central role in negatively regulating the tiller angle^20^.

Plant architectures are also significantly associated with plant hormones, including auxin, gibberellic acid (GA), and strigolactones. GA have long been thought to promote upward growth and inhibit bending in Japanese cherry^21–23^. Moreover, *gibberellin 3β-hydroxylase* (*GA3ox*) expression levels in weeping-type Japanese cherry were shown to be higher than those in upright-type Japanese cherry^24^. *GA3ox1* is involved in the final step of GA biosynthesis and loss of *GA3ox1* function leads to prostrate and dwarfed growth^25^. It has also been suggested that GA function in gravitropic responses, although GA1 asymmetry does not trigger gravitropic responses^25^. Nugroho et al.^26^ suggested that GA has a strong influence on the initial stages during the formation of tension wood and stem gravitropism in *Acacia mangium*. In peach, the *pl* phenotype may be associated with GA signaling and upright branch internodes are shorter than those of weeping branches in peach, implying that the upright type may have a lower GA content^27^. Transcriptional results suggest that GA and auxin play a vital role in controlling the weeping trait in *Salix matsudana*^28^. Transcriptome analysis of columnar apple trees showed that genes associated with auxin and GA, such as *xyloglucan endotransglycosylase* (*XTH*) and *gibberellin 2-β-dioxygenase 1* (*GA2ox1*), are located in the *Co* region linked with the apple column trait^29^. Previous research also suggested that GA acting either alone or with auxin promote secondary xylem formation^22,30,31^.

Based on a prior analysis of weeping traits, many genes related to phytohormones, light, and gravity may be responsible for growth orientation. However, the potential molecular basis of weeping traits in woody plants remains poorly defined^27^. Using sequencing technology, the mechanisms of a model species can be simultaneously compared, which is more conducive to the analysis of woody plant architecture mechanisms. *Lagerstroemia*, which exhibits various plant architectures, has a large number of flowers and a long flowering period. Moreover, most of its seedlings with short juvenile periods grow rapidly, as do flowers with an age of over 1 year^32^. Therefore, *Lagerstroemia* is widely used in gardens and has specific economic benefits^33^. Research on the plant architecture of crape myrtle is mainly focused on dwarf traits. Two markers that are highly correlated with internode length and one marker that is highly correlated with primary lateral branch height have been validated in crape myrtle^34^. The analysis of transcriptome and hormone levels in dwarf and non-dwarf crape myrtles suggested that internode length is controlled by an interaction between auxin and GA4^32^. The weeping plants examined in our study are dwarf plants with weeping branches. However, the molecular mechanism underlying the weeping trait in crape myrtle has not been elucidated. After examining the phenotype and histology of these plants, and performing GA3 treatment, we used sequencing technology combined with weighted gene coexpression network analysis (WGCNA) to obtain candidate genes. Subsequently, virus-induced gene silencing (VIGS) was used to verify the functions of the candidate genes. Studying the potential mechanisms that result in the weeping phenotype of crape myrtle will be helpful for breeding cultivars suitable for weeping culture. The results of this study may provide valuable information for the further analysis of the molecular mechanism of the weeping trait in woody plants.

## Materials and methods

### Plant materials

Weeping and upright progenies of a BC_1_ population were derived from the backcross of a weeping F_1_ individual (♀) and *Lagerstroemia fauriei* (♂), which was described in our previous studies^35^. The F_1_ population was generated from upright *L. fauriei* (♀) and weeping *Lagerstroemia indica* “Creole” (♂). The BC_1_ population consisted of 174 progenies. Ten extreme upright progenies and ten extreme weeping progenies were selected from the BC_1_ population and were propagated by cutting for use in this study. The BC_1_ population and cutting seedlings were grown in a greenhouse at Beijing Forestry University.

The transcriptome samples included ten upright lines and ten weeping lines (Supplementary Table S1). Three individuals of each line, for a total of 30 individuals, were mixed into 1 sample, which was repeated 3 times during different developmental stages. The samples in the first group (group A) reflected the initial emergence of the axillary bud. The samples in the second and third groups consisted of axillary shoots and young stems, respectively, with four internodes. The second and third groups were named groups B and C, respectively. Several organs from the upright and weeping lines, including the axillary shoots, stem1 (from the apical first to second internodes), stem2 (from the apical third to fourth internodes), leaves, and roots, were used to examine tissue specificity. Axillary buds/axillary shoots and stems (from the apical first to second internodes) were collected at the zero (T0)-, two (T1)-, four (T2)-, and eight (T3)-internode stages. These samples were employed to investigate the expression patterns at four developmental stages. Plant samples for RNA-sequencing (RNA-seq) and quantitative real-time PCR were immediately frozen in liquid nitrogen, and then stored in a −80 °C refrigerator.

### Paraffin sections

The semi-lignified stems from the upright and weeping lines were placed in FAA solution (formaldehyde : glacial acetic acid : 70% ethanol = 1 : 1 : 18 v/v). After rinsing, the samples were dehydrated in a gradient ethanol series (70%, 85%, 95%, and 100% alcohol for 1 h each). Then, the samples were embedded in paraffin. Sections of 8 μm in thickness were applied to silane-coated glass slides and the paraffin was removed. A gradient ethanol series was employed to dehydrate the sections. Fast green and the counterstain safranin were employed to stain the sections. Finally, the sections were observed on an automatic digital slide scanner (Panoramic MIDI, 3DHISTECH Ltd, Budapest, Hungary).

### Phenotypic characterization and GA3 treatment

We measured seven phenotypic traits in the extreme weeping and upright progenies in the BC1 population. The measured traits included plant height, plant width, plant canopy angle, the branching angle of the main branch, branching height, leaf length, and leaf width. Plant height and branching height were measured from the base of the soil surface to the highest point. The branching angle of the main branch corresponds to the angle between the main branch and the vertical direction. The branching canopy angle is the sum of the angles of inclination at the widest position of the canopy from the vertical orientation on both sides. The measurement methods were described in a previous study^35^. To investigate the growth direction of branches, we examined 1-year-old cutting seedlings sprayed with 150 mg/L GA3 or H_2_O every 3 days between 22 May 2019 and 6 June 2019. Six upright individuals and six weeping individuals were sprayed as one treatment.

### Total RNA isolation and cDNA synthesis

Total RNA was extracted using a Plant RNA Kit (Omega Bio-Tek, Doraville, GA, USA) and then used to construct cDNA libraries using the Dynabeads™ mRNA Purification Kit (Thermo Fisher Scientific, Wilmington, DE, USA) for RNA-seq. cDNA for quantitative reverse-transcriptase PCR (qRT-PCR) and clones were synthesized using the PrimeScript RT reagent Kit (TaKaRa, Dalian, China). All experimental steps were performed according to the manufacturer’s manual.

### Transcriptomic analysis

Eighteen cDNA libraries were constructed and sequenced on the BGISEQ-500 platform after mRNA enrichment, RNA fragmentation and reverse transcription, end repair, poly A tail addition and adaptor ligation, PCR amplification, denaturation, and cyclization (BGI, Qingdao, China). The read length corresponded to a 100 bp paired-end layout. To obtain clean reads, adapter and low-quality sequences were removed from the raw RNA-seq reads. We used Trinity to perform de novo assembly with clean reads and then used Tgicl to cluster the transcripts to unigenes. The unigenes were divided into two types as follows: (1) clustered, with the prefix CL followed by the cluster id (each cluster consists of several unigenes for which the similarity between the unigenes is >70%), and (2) singletons, with the prefix unigene. A unigene represents a transcript. To annotate gene functions, BLASTn was used to retrieve the nucleotide sequence database (NT) annotations. Nonredundant protein sequence database (NR), clusters of orthologous groups for eukaryotic complete genomes (KOG), Kyoto Encyclopedia of Genes and Genomes (KEGG), and SwissProt annotations were collected by using BLASTx and Diamond. InterPro annotations were collected with InterProScan5^36^. Blast2GO^37^ and NR annotations were used to identify the Gene Ontology (GO) annotations.

The clean reads were mapped to the unigenes with Bowtie2^38^. Gene expression levels were calculated using RNA-seq by expectation maximization^39^. Three biological replicates were used to control the accuracy in the calculation of gene expression levels. The differentially expressed genes (DEGs) were detected with EBSeq^40^. Unigenes were considered to be significantly differentially expressed if the fold change was ≥2.00 and the posterior probability of equivalent expression was ≤0.05. GO and KEGG enrichment analyses of the DEGs were performed by using the phyper function in R. The initial *p*-values were corrected according to the false discovery rate (FDR), and GO terms and KEGG pathways with an FDR < 0.05 were considered to be significantly enriched.

The WGCNA package in R was used to identify modules of closely related genes based on the gene expression levels^41^. The DEGs from groups A, B, and C were used for analysis. First, a similarity matrix was produced by computing the correlations between all the included genes; genes with a low coefficient were then removed. Weighted coexpression network construction was performed based on the remaining genes. The network construction parameters were as follows: weight = 0.6 and min module size = 20. The coexpression gene network results were visualized in Cytoscape (v3.3.0). To find the target module among the coexpression gene networks, GO and KEGG enrichment analysis of each module was performed.

The raw sequence data reported in this study have been deposited in the Genome Sequence Archive^42^ in the BIG Data Center^43^ (Beijing Institute of Genomics (BIG), Chinese Academy of Sciences) under accession number CRA001957, which is publicly accessible at https://bigd.big.ac.cn/gsa.

### Expression pattern analysis

Based on the transcriptomic analysis, qRT-PCR primers (Supplementary Table S2) were designed by Integrated DNA Technologies (https://sg.idtdna.com/scitools/Applications/RealTimePCR/). The qRT-PCR assay was performed using TB Green® Premix Ex Taq™ II (TaKaRa) in a CFX Connect Real-Time System (Bio-Rad Laboratories, Inc., Hercules, CA, USA) according to the manufacturer’s protocol. The *elongation factor-1-alpha* (*EF1α*) gene^44^ was used as a reference gene for the normalization of gene expression. The 2−ΔΔCt method was used to calculate gene expression^45^. Three biological replicates and three technical replicates were performed to control the accuracy. SPSS 20.0 (SPSS, Inc., Chicago, IL, USA) was used to test the significant differences between the controls and treatments.

### VIGS assay in crape myrtle

The plasmid vectors pTRV1 and pTRV2, which encode the RNA1 and RNA2 genomes of tobacco rattle virus (TRV), respectively, were used for the preparation of TRV vectors^46^. The pTRV2:*LfiGRAS1* vector included a specific 210 bp fragment from *LfiGRAS1*, which was cloned from a cDNA template from the axillary shoot of a cutting seedling using gene-specific primers (Supplementary Table S3). The pTRV2:*LfiGRAS1* plasmid was confirmed by sequencing and then transformed into chemically competent *Agrobacterium* strain GV3101 cells using a liquid nitrogen freezing and thawing method. VIGS was performed in crape myrtle based on a previous protocol^47^. Three, three, and six weeping plants were used as the vector control, non-treated weeping plants, and *LfiGRAS1*-silenced plants, respectively. The pTRV2:*LfiGA2ox* vector included a specific 306 bp fragment from *LfiGA2ox* and the pTRV2:*LfiGRAS2* vector included a specific 190 bp fragment from *LfiGRAS2*. The other steps were the same as for pTRV2:*LfiGRAS1*.

## Results

### Phenotypic analysis of upright and weeping crape myrtle

**Fig. 1:**
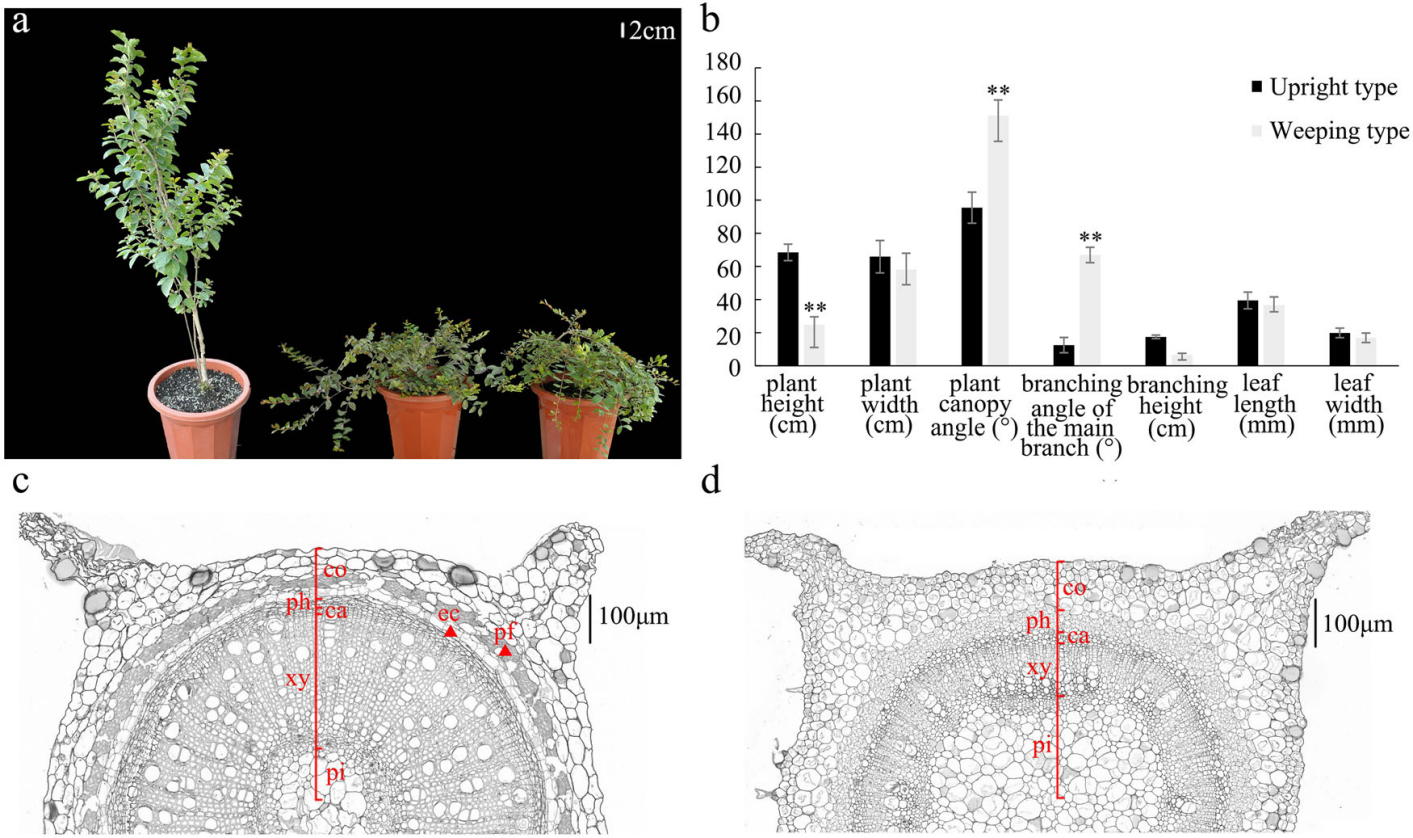
Phenotypic analysis and paraffin sections of upright and weeping individuals. **a** Extremely upright and extremely weeping progenies in the BC_1_ population. **b** Analysis of seven phenotypic traits from ten extremely upright and ten extremely weeping progenies in the BC_1_ population. **A significant difference at *P* < 0.01 between the two types as determined according to the *t*-test. **c** Paraffin section of a stem from an upright line. **d** Paraffin section of a stem from a weeping line. ca, cambium; co, cortex; ec, endodermal cells; pf, phloem fiber; ph, phloem; pi, pith; xy, xylem.

Among upright and weeping individuals, the plant height, plant canopy angle, and branching angle of the main branch were significantly different (Fig. 1a, b). The average plant height of weeping individuals was 24.65 cm, whereas the average plant height of upright individuals was 68.5 cm. Therefore, the weeping plants used in this study were characterized by weeping and dwarfing (Fig. 1a). The weeping trait was mainly determined during the early development of shoots (Supplementary Fig. S1a, b). In the paraffin sections, upright individuals exhibited obvious endodermal cells and phloem fibers, whereas the weeping plants did not (Fig. 1c, d). This result indicated that endodermal cells and phloem fibers may be responsible for the abnormal weeping trait phenotypes.

**Fig. 2:**
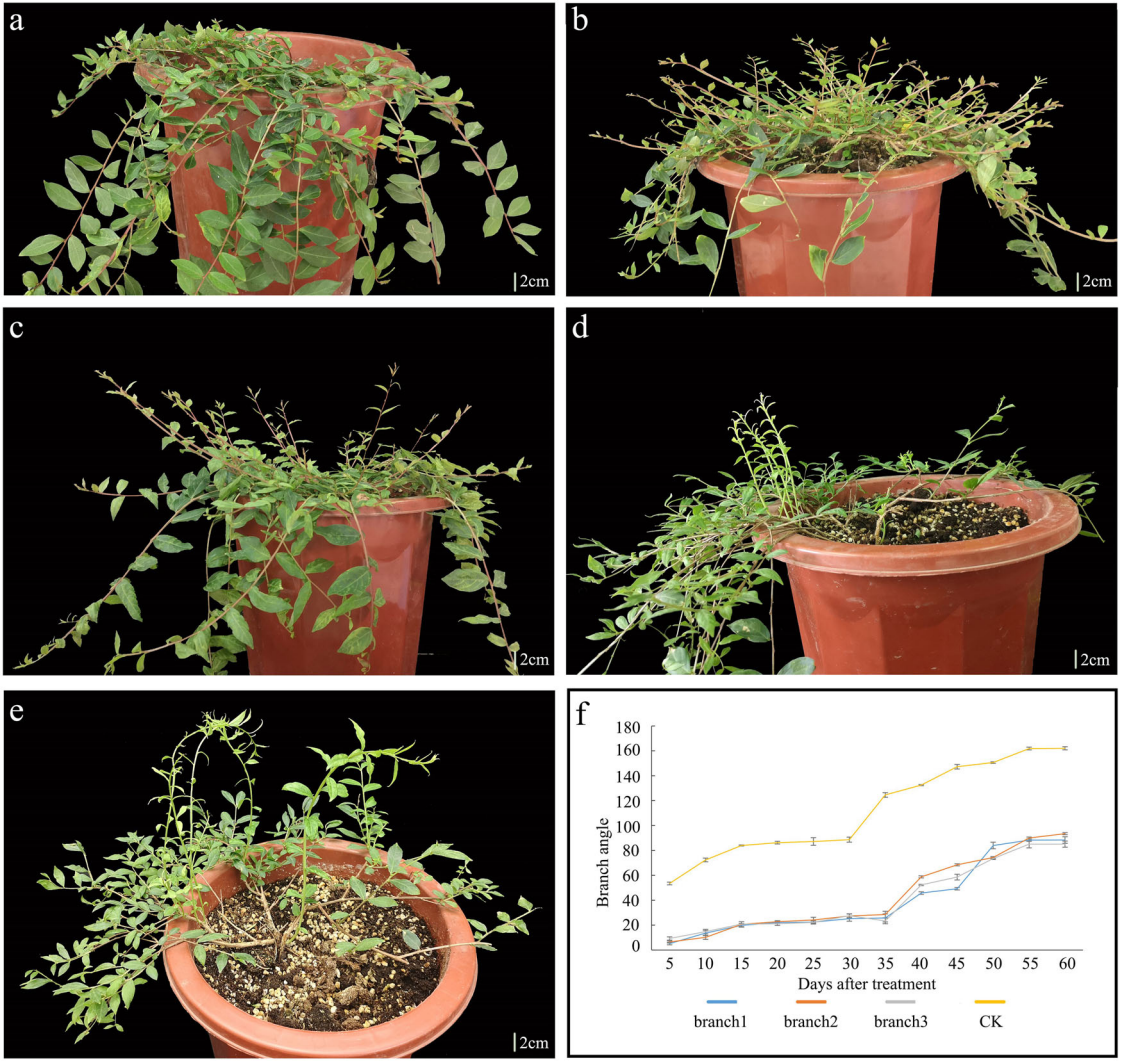
GA3 treatment of the weeping lines. **a** Weeping lines after treatment with H_2_O. **b**, **d** Two weeping individuals 20 days after treatment with 150 mg/L GA3. **c** A weeping individual 35 days after treatment with 150 mg/L GA3. **e** Weeping individuals 45 days after treatment with 150 mg/L GA3. **f** Branching angle trend after GA3 treatment. “branch1,” “branch2,” and “branch3” are branches from the different weeping lines after treatment with 150 mg/L GA3. “CK” represents branches from the weeping lines after treatment with H_2_O.

After exogenous GA3 treatment, the new shoots from weeping individuals grew in a negatively geotropic manner and inhibited bending (Fig. 2a–e). As the branches grew, they gradually bent to elongate downward and the branch angle gradually increased (Fig. 2e, f). However, the growth direction of the previous branches did not change dramatically. The upward growth of some branches was only slightly promoted. No differences in the branching angle were found in upright individuals treated with exogenous GA3. These results strongly suggested that GA3 might be the key factor determining the branching angle and promoting the development of upright plants.

### De novo genome assembly and annotation

To dissect the molecular mechanism underlying the weeping phenotype, axillary buds, axillary shoots, and stems from the upright and weeping lines were subjected to RNA-seq analysis. In total, 151.76 Gb of raw reads were generated by sequencing on the BGISEQ-500 platform. After the removal of adapter and low-quality sequences, more than 93% clean reads were obtained from each sample. We identified 171,939 unigenes (Supplementary Table S4) by assembling all the samples together and filtering for abundance. The total length, average length, N50, and GC contents of the unigenes were 242,165,937 bp, 1,408 bp, 2,097 bp, and 46.04%, respectively (Supplementary Table S5). Seven functional databases were used to annotate the unigenes. Finally, 121,212 (NR: 70.50%), 54,160 (NT: 31.50%), 86,605 (SwissProt: 50.37%), 98,871 (KOG: 57.50%), 94,206 (KEGG: 54.79%), 97,375 (InterPro: 56.63), and 3814 (GO: 2.22%) unigenes were annotated (Supplementary Fig. S2a and Supplementary Table S6). Based on the functional annotation results from the NR database, we calculated the ratios of different species according to the unigene annotation and drew a distribution map (Supplementary Fig. S2b). In GO clustering, 7235, 9027, and 4584 unigenes were classified into the biological process, cellular component, and molecular function categories, respectively (Supplementary Fig. S2c). In the KEGG pathway analysis, 94,206 annotated unigenes were classified into 138 pathways (Supplementary Fig. S2d). In the KOG functional classification, 98,871 annotated unigenes were classified into 25 functional groups (Supplementary Fig. S2e). The principal component analysis showed good biological duplication among the samples (Supplementary Fig. S2f).

### DEGs and functional enrichment analysis

The three groups (groups A, B, and C) presented many DEGs. A comparison of all the DEGs in the three groups indicated that 12,044 unigenes were differentially expressed in the three groups (Supplementary Fig. S3a). The unigenes that were only differentially expressed in group A may be associated with the early development of the weeping trait. There were 3107 DEGs that fell within the intersection of groups B and C. These 3107 DEGs were expressed during the second period (the four-internode stage). The 12,044 DEGs included in the 3 groups were mainly assigned to global and overview maps, translation, carbohydrate metabolism, folding, sorting and degradation, and signal transduction via KEGG analysis (Supplementary Fig. S3b). Similarly, the DEGs from groups A, B, and C were mainly assigned to five levels in the KEGG pathway analysis (Supplementary Fig. S4a, b, c).

The top 20 enriched pathways were used to draw the enrichment map. For group A (Supplementary Fig. S4d), KEGG enrichment analysis identified 20 enriched pathways (*P* < 0.05). These pathways belonged to the metabolism, genetic information processing, or organismal systems levels. In total, 1113 DEGs were classified into the plant hormone signal transduction category. Focusing on group B (Supplementary Fig. S4e), the most enriched KEGG pathway was a circadian rhythm plant (370 unigenes). Another 11 pathways were also significantly enriched (*P* < 0.05) in group B. For example, the plant hormone signal transduction pathway included 990 DEGs (*p* = 0.0399). In group C (Supplementary Fig. S4f), 11 pathways were significantly enriched (*P* < 0.05). Similar to group A, these pathways all belonged to the metabolism, genetic information processing, and organismal systems levels. As shown for the enriched KEGG pathways from group C, the plant hormone signal transduction pathway was also relatively important.

### Identification of WGCNA modules

**Fig. 3:**
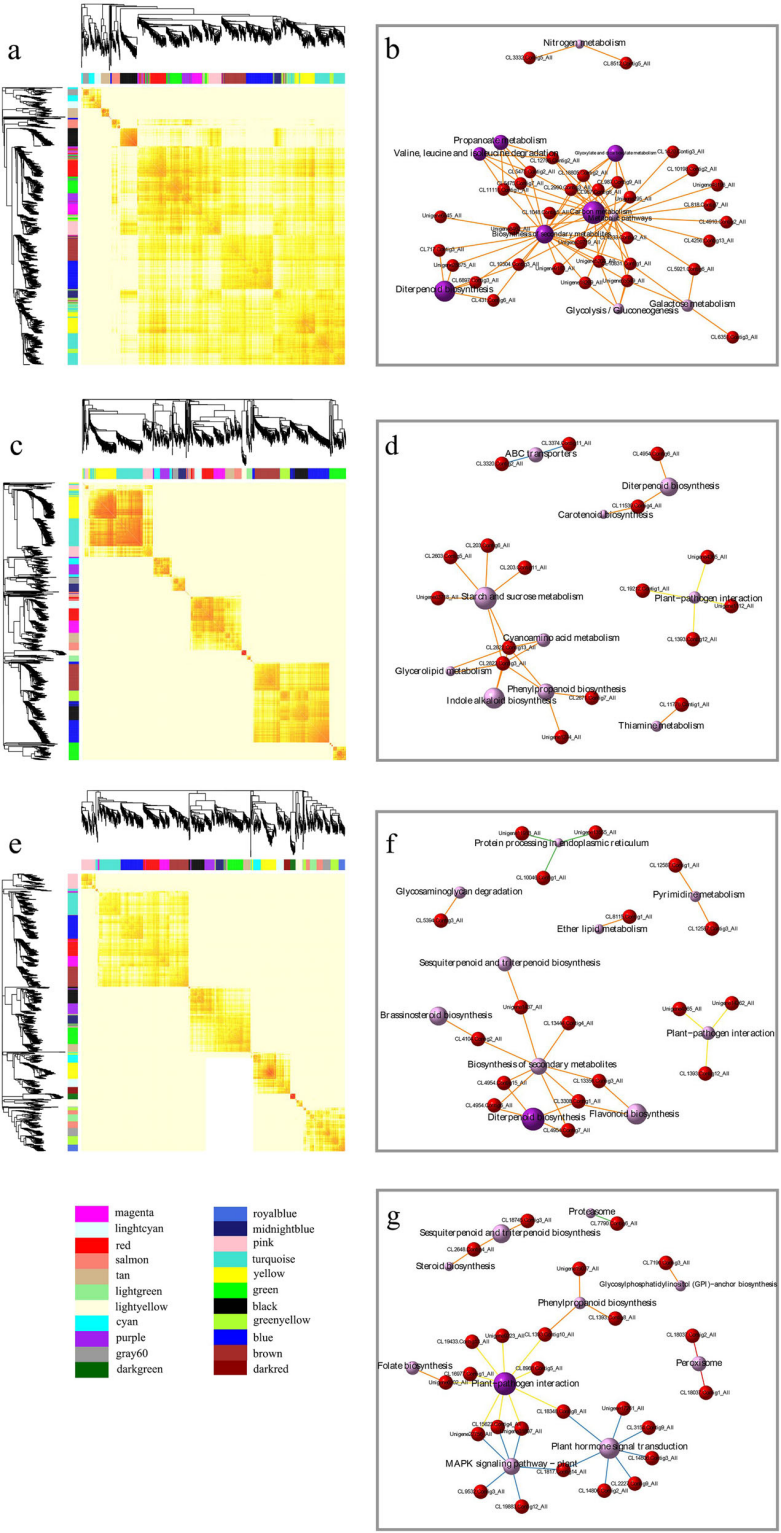
Construction of the WGCNA and KEGG functional analysis of the four core modules. **a** Division and heatmap of the modules in group A. **b** Gene network based on KEGG pathway enrichment for “turquoise” in group A (M1). **c** Division and heatmap of modules in group B. **d** Gene network based on KEGG pathway enrichment for “green” in group B (M2). **e** Division and heatmap of modules in group C. **f** Gene network based on KEGG pathway enrichment for “black” in group C (M3). **g** Gene network based on KEGG pathway enrichment for “turquoise” in group C (M4).

Using the DEGs from groups A, B, and C, three gene coexpression networks were constructed. The coexpression gene network analysis of the A, B, and C groups identified 17, 19, and 22 distinct modules, respectively (Fig. 3a, c, e). The largest module (the “turquoise” in group B) contained 147 unigenes, whereas the smallest module (“gray 60” in group A and “light yellow” in group B) contained 23 unigenes.

To identify the core modules, we performed GO term enrichment analysis, KEGG enrichment analysis, and module and sample correlation analysis (Supplementary Fig. S5). Four core modules (“turquoise” in group A (M1), “green” in group B (M2), “black” (M3) and “turquoise” (M4) in group C) were identified through correlation analysis. M1, M2, M3, and M4 consisted of 134, 76, 66, and 106 DEGs (Supplementary Table S7), which were classified into 44, 29, 29, and 25 KEGG pathways, respectively. The diterpenoid biosynthesis pathway was significantly enriched (*P* < 0.05) in M1, M2, and M3 (Supplementary Fig. S6a, b, c). Plant hormone signal transduction was significantly enriched (*P* < 0.05) in M1 and M4 (Supplementary Fig. S6a, d). The KEGG pathway enrichment network for M1, M2, M3, and M4 also indicated that the diterpenoid biosynthesis and plant hormone signal transduction pathways were key pathways in the network (Fig. 3b, d, f, g). All DEGs in the diterpenoid biosynthesis pathway from M1, M2, M3, and M4 were involved in GA synthesis. Thus, we focused on GA synthesis and signal transduction in subsequent studies.

### Analysis of DEGs involved in GA synthesis and signal transduction

The results of GA3 treatment showed that GA played important roles in the weeping trait. Moreover, transcriptome analysis showed that GA synthesis and the associated signal transduction pathway played a significant role in the branching of crape myrtle. Therefore, the expression levels of transcripts involved in GA synthesis and signal transduction were analyzed.

**Fig. 4:**
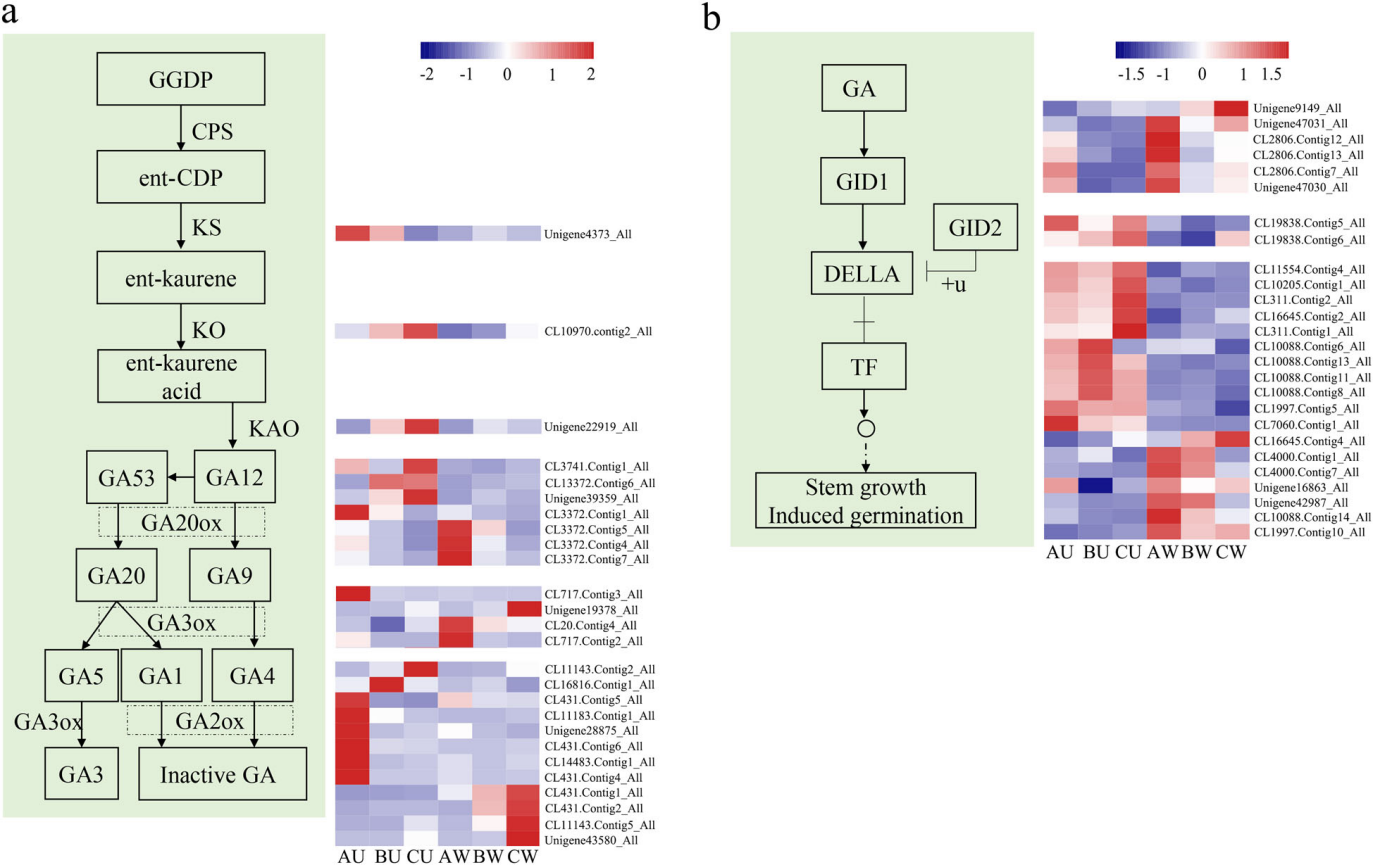
Analysis of DEGs involved in GA synthesis and signal transduction. **a** Analysis of DEGs involved in GA synthesis. **b** Analysis of DEGs involved in GA signal transduction. Abbreviations: AU, upright type in group A; AW, weeping type in group A; BU, upright type in group B; BW, weeping type in group B; CU, upright type in group C; CPS, ent-copalyl diphosphate synthase; CW, weeping type in group C; ent-CDP, ent-copalyl diphosphate; GA20ox, gibberellin 20 oxidase; GA3ox, gibberellin 3-β-dioxygenase; GA2ox, gibberellin 2-β-dioxygenase; GGDP, geranylgeranyl-PP; KAO, ent-kaurenoic acid oxidase; KS, ent-kaurene synthase; KO, ent-kaurene oxidase.

To synthesize GA, six vital enzymes, including CPS (ent-copalyl diphosphate synthase), KS (ent-kaurene synthase), KO (ent-kaurene oxidase), KAO (ent-kaurenoic acid oxidase), GA20ox (gibberellin 20 oxidase), and GA3ox (gibberellin 3-β-dioxygenase), were employed. In addition, GA2ox was used to degrade active GA. Three biosynthetic genes, *LfiKS*, *LfiKO*, and *LfiKAO*, were significantly downregulated in the weeping lines (Fig. 4a). The transcriptional levels of the three genes in the upright lines were at least 2.5-fold higher than those in the weeping lines. This result indicated that the contents of GA in upright *Lagerstroemia* may be higher than those in weeping plants. Three *GA20ox* transcripts (CL13372.Conting4_All, CL13372.Conting5_All, and CL13372.Conting7_All) were highly expressed in the axillary buds in the weeping lines and expressed at low levels in the axillary shoots and stems in the weeping and upright lines (Fig. 4a). Both CL13372.Conting1_All (*GA20ox*) and CL717.Conting3_All (*GA3ox*) were only highly expressed in the axillary buds in the upright lines (Fig. 4a). The same property was observed for six *GA2ox* transcripts. These transcripts may play an important role in controlling plant architecture during the early stage. There were transcripts with similar functions that were highly expressed in axillary shoots or stems, which may function in later stages. The GA receptor GID1, F-box protein GID2, and DELLA protein were employed as GA signal transduction components together. The transcriptome included transcripts that were annotated as *GID1*, *GID2*, and *DELLA*. A number of these transcripts showed significantly differential expression in the upright and weeping lines (Fig. 4b). We selected three genes for follow-up studies based on their expression levels and fold changes. We designated CL11143.Conting5_All, CL10088.Conting14_All, and Unigene 16863 as *LfiGA2ox*, *LfiGRAS1*, and *LfiGRAS2*, respectively.

### Expression pattern analysis of gibberellin-related genes

**Fig. 5:**
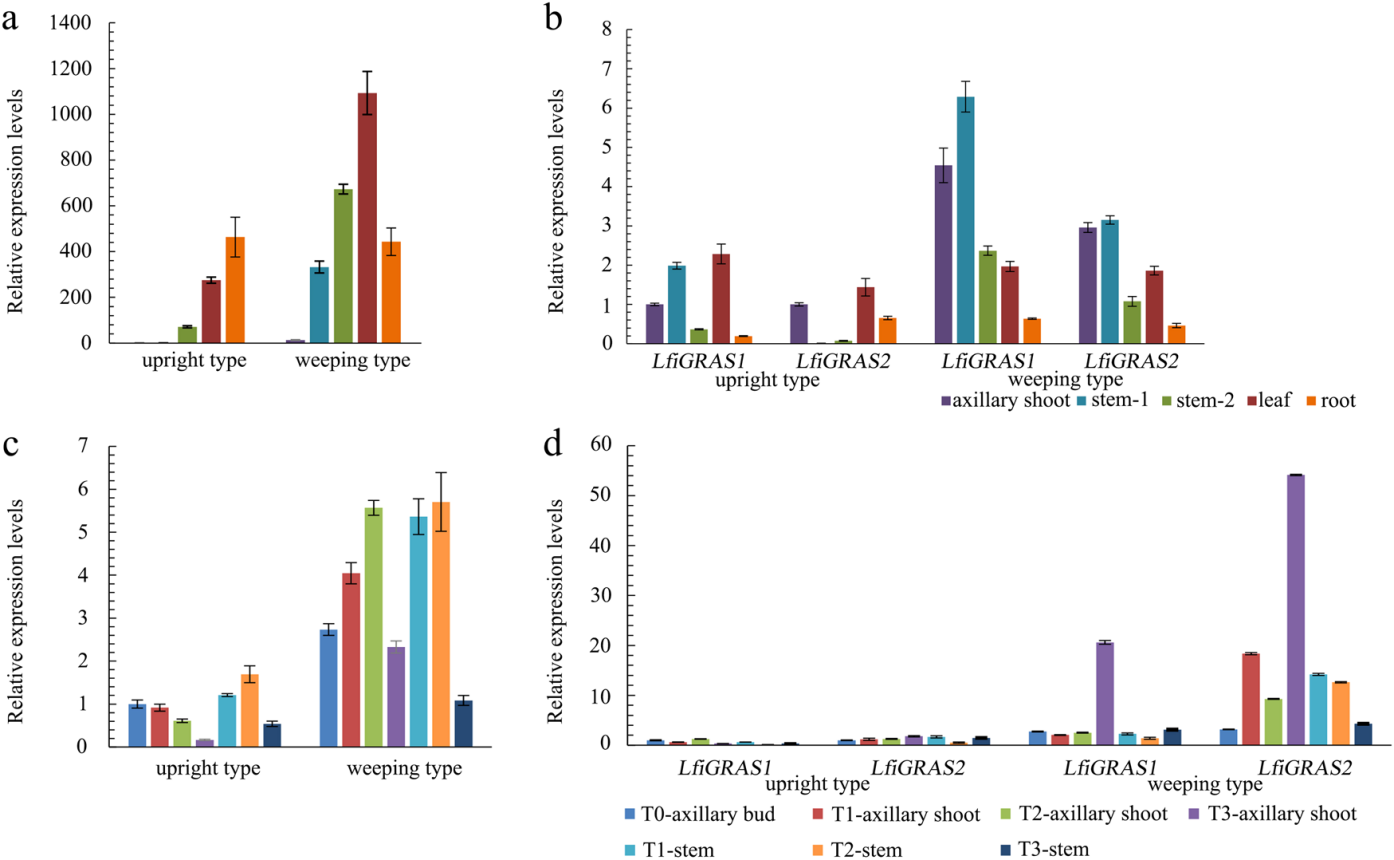
Spatiotemporal expression analysis of *LfiGA2ox*, *LfiGRAS1*, and *LfiGRAS2*. **a** Spatial expression analysis of *LfiGA2ox*. **b** Spatial expression analysis of *LfiGRAS1* and *LfiGRAS2*. **c** Expression analysis of *LfiGA2ox* in different growth stages. **d** Expression analysis of *LfiGRAS1* and *LfiGRAS2* in different growth stages. stem1: from the apical first to second internodes. stem2: from the apical third to fourth internodes. T1: the two-internode stage. T2: the four-internode stage. T3: the eight-internode stage. Each bar represents the mean values of three technical repetitions and three biological replicates. Error bars were obtained from three biological replicates.

Five tissues, the axillary shoots, stem1 (from the apical first to second internodes), stem2 (from the apical third to fourth internodes), leaves, and roots, were used to examine the tissue specificity of *LfiGA2ox*, *LfiGRAS1*, and *LfiGRAS2* by qRT-PCR. In weeping plants, *LfiGA2ox* expression levels in the leaves were surprisingly high, although they were lower in the axillary shoots (Fig. 5a). Regardless of the location in the axillary shoot, stem, axillary bud, or leaf, *LfiGA2ox* expression was significantly lower in the upright lines than in the weeping lines. *LfiGRAS1* was transcribed in each examined tissue (Fig. 5b). In the weeping lines, stem1 and the axillary shoots possessed high expression levels. *LfiGRAS1* expression in stem2 was considerably lower than that in stem1. *LfiGRAS1* was not highly expressed in the roots or leaves. In the axillary shoots, stem1, stem2, and roots, *LfiGRAS1* expression was higher in the weeping lines than in the upright lines. A similar situation was not observed in the leaves. *LfiGRAS2* was also expressed in every tested organ (Fig. 5b). In the stems of the upright lines, *LfiGRAS2* expression was very low (Fig. 5b, d). In the weeping lines, *LfiGRAS2* expression was the lowest in the roots.

We next tested the expression levels of *LfiGA2ox*, *LfiGRAS1*, and *LfiGRAS2* in different growth stages. During the early stage of axillary bud outgrowth, *LfiGA2ox* levels gradually increased in the weeping lines (Fig. 5c). When the stems had extended to a certain level, *LfiGA2ox* levels began to decrease. In the weeping lines, the axillary shoots possessed particularly high *LfiGRAS1* expression levels when the number of internodes was eight (Fig. 5d). The expression trend for *LfiGRAS1* in the axillary shoots was different from that in the stems. As the stems elongated, *LfiGRAS1* expression in the axillary shoots gradually increased. However, in the stems, the expression levels first decreased and then increased. At every stage, *LfiGRAS1* and *LfiGRAS2* were transcribed at high levels in the weeping lines but at low levels in the upright lines. At the eight-internode stage, *LfiGRAS2* was also expressed at high levels in the axillary buds in the weeping lines.

### VIGS in crape myrtle

**Fig. 6:**
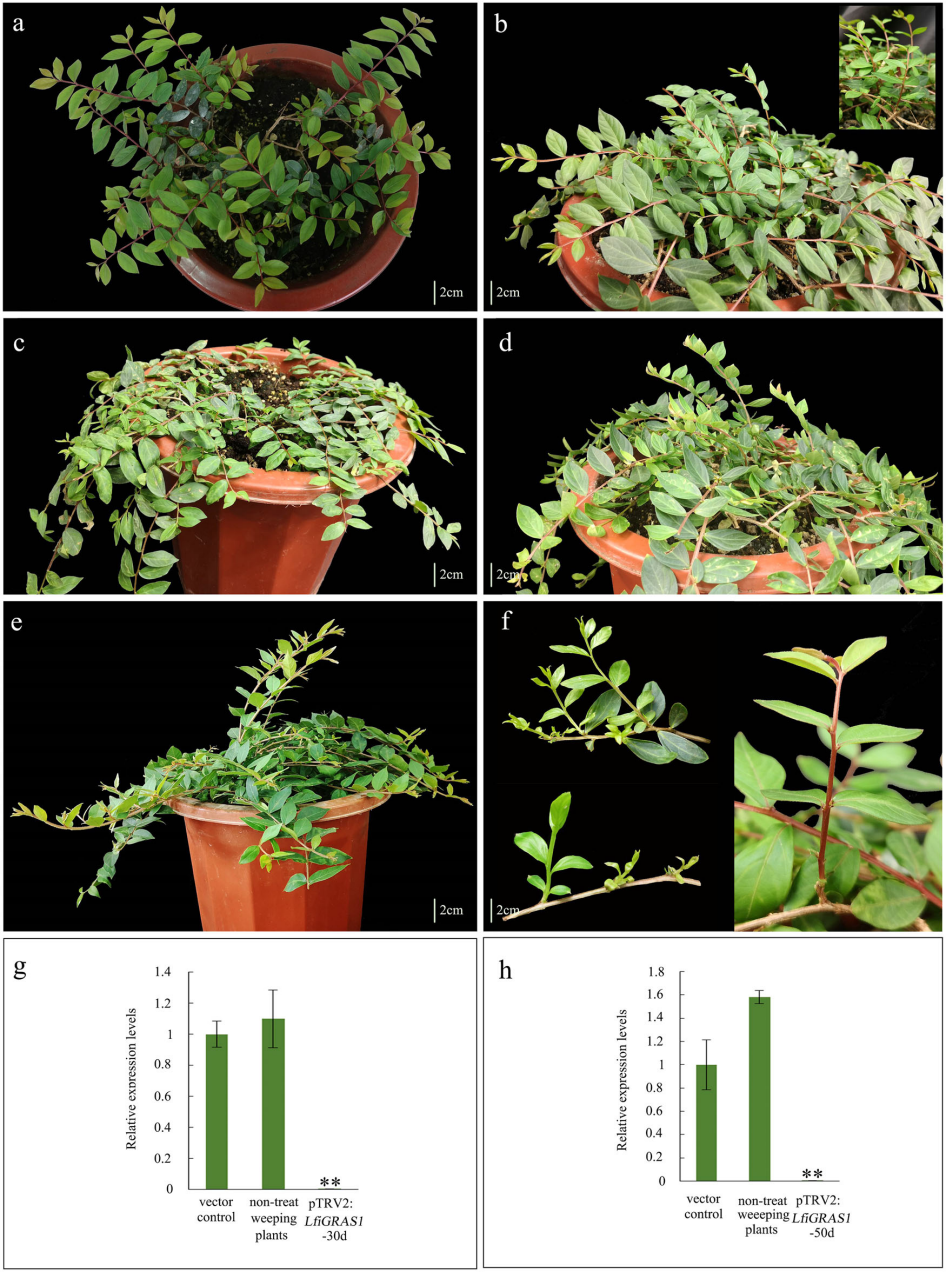
Growth phenotype and expression analysis of *LfiGRAS1*-silenced plants. **a** Non-treated weeping plant. **b** *LfiGRAS1*-silenced plants 30 days after infection. **c** Vector control plant (infected by pTRV1 and pTRV2). **d** *LfiGRAS1*-silenced plants 50 days after infection. **e** *LfiGRAS1*-silenced plants. **f** Branches from *LfiGRAS1*-silenced plants. **g** Expression analysis of *LfiGRAS1* 30 days after infection. **h** Expression analysis of *LfiGRAS1* 50 days after infection. Asterisks indicate that the gene was significantly upregulated or downregulated according to the *t*-test (*P < 0.05 and **P < 0.01).

VIGS was used to reduce the expression of *LfiGA2ox*, *LfiGRAS1*, and *LfiGRAS2* to further elucidate the function of the three genes in the weeping trait. Maps of the silencing fragment constructs for the genes used in VIGS are shown in Supplementary Fig. S7. qRT-PCR was performed on newly grown axillary shoots from *LfiGRAS1*-infected plants, vector controls (Fig. 6c), and non-treated weeping plants (Fig. 6a) at 30 and 50 days after infection. The results showed that *LfiGRAS1* expression levels were reduced (Fig. 6g, h). Thirty days after infection, *LfiGRAS1* expression levels in the *LfiGRAS1*-silenced plants were downregulated ~320-fold compared with those in the vector controls and ~350-fold compared with non-treated weeping plants. Fifty days after infection, the transcript levels in *LfiGRAS1*-silenced plants were still considerably lower than those in the vector controls and non-treated weeping plants. These results indicated that silencing was highly efficient.

We continued observing the plant phenotype from 15 to 60 days after infection. Three of the six infected plants showed a relevant phenotype. We found that reducing the expression of *LfiGRAS1* could rescue or delay the bending of some new branches in the three infected plants (Fig. 6b, d, e, f and Supplementary Fig. S10). The vector controls and non-treated weeping plants showed significant weeping before reaching the three-internode stage. The branching angles of the vector controls and all non-treated weeping plants were >65°. The *LfiGRAS1*-silenced plants showed many branches with a branch angle of <40°. The branching angle of some branches even approached that in the upright plants. The *LfiGRAS1*-silenced plants also showed upward growth at the six-internode stage. With the elongation of the stem, the shoot began to adopt a slightly weeping appearance. In addition, compared with the vector controls and non-treated weeping plants, the *LfiGRAS1*-silenced plants exhibited many branches that showed an upward growth trend.

Thirty days after infection, *LfiGA2ox* expression levels in the *LfiGA2ox*-silenced plants were downregulated ~16-fold compared with those in the vector controls and ~19-fold compared with those in the non-treated weeping plants (Supplementary Fig. S9). *LfiGRAS2* expression levels in the *LfiGRAS2*-silenced plants were downregulated ~12-fold compared with those in the vector controls and ~19-fold compared with those in the non-treated weeping plants (Supplementary Fig. S10). Compared with the vector controls and the non-treated weeping plants, the *LfiGA2ox*-silenced plants had more axillary buds and the *LfiGRAS2*-silenced plants exhibited many malformed branches (Supplementary Figs. S9 and S10).

## Discussion

Plant architecture has a powerful effect on the productivity of orchards and forestry plantations. Research on plant architecture helps humans manipulate branch numbers, branch orientations, and tree size. An ideal plant architecture could minimize the need for pruning and maximize light penetrance. In recent years, weeping plants have received attention because of their beautiful shape. However, the potential molecular mechanisms determining weeping in crape myrtle have not been elucidated, despite their significance.

Based on our preliminary study, there may be other regulatory mechanisms underlying the weeping trait in *Lagerstroemia*. To reveal the underlying mechanism of the weeping trait, phenotypic measurements, paraffin section observations, and exogenous GA3 treatment were performed in weeping and upright individuals. The results indicated that the weeping plants lacked endodermal cells and phloem fibers, and that GA3 treatment promoted new shoot growth in a negative geotropic orientation. Endodermal cells in the shoots are the site of gravity sensing in dicotyledonous plant branches^48^. The absence of a normal endothelial layer in the *shoot gravitropism* (*sgr7*)/*short-root* (*shr*) and *shoot gravitropism 1* (*sgr1*)/*scarecrow* (*scr*) *Arabidopsis thaliana* mutants leads to an absence of gravitropism in the shoots^49,50^. The *weeping* (*we1*) gene, which is an ortholog of *Arabidopsis SCR*, and *weeping2* (*we2*), an ortholog of *Arabidopsis SHR*, were identified in a gravitropic Japanese morning glory mutant with defective endodermal cells^51,52^. Exogenous GA treatment was found to rescue prostrate ryegrass mutants to a wild-type phenotype, implying that the prostrate and dwarf phenotypes are both caused by GA deficiency^53^.

To dissect the molecular mechanism underlying the weeping phenotype in crape myrtle, RNA-seq was performed. Transcriptome data were verified using qRT-PCR (Supplementary Fig. S11). On the basis of the functional analysis of DEGs and WGCNA, we suggest that GA synthesis and signal transduction pathways may strongly impact weeping traits. Some genes involved in GA synthesis and signal transduction were mined. VIGS was designed to investigate the functions of the three candidate genes. The injection of crape myrtle with the upright trait caused the branches to bend downwards. The VIGS phenotype accurately mimicked that of crape myrtle with the weeping trait. Therefore, the injection of weeping branches requires the selection of genes that are highly expressed in weeping lines. Based on expression levels and fold changes, *LfiGA2ox*, *LfiGRAS1*, and *LfiGRAS2* were selected. The VIGS results indicate that *LfiGA2ox*, *LfiGRAS1*, and *LfiGRAS2* have different functions, among which *LfiGRAS1* may be related to the weeping trait in the crape myrtle.

*GA2ox* is responsible for irreversibly inactivating bioactive GA or is precursors via 2-β hydroxylation^54,55^. GA stimulates the outgrowth of axillary buds in peach and *Jatropha curcas*^56,57^. The overexpression of *GA2ox1* in poplar resulted in a reduction in the contents of the main bioactive components GA1 and GA4. In addition, the phenotype was dwarfed and showed a decrease in the branch number^58^. Similarly, a reduction in *LfiGA2ox* expression levels promoted the outgrowth of axillary buds in crape myrtle (Supplementary Fig. S9). Interestingly, this reduction also greatly promoted the germination of buds on old stems. However, the overexpression of *GA2ox* led to dwarfism and greater tiller numbers in rice and switchgrass (*Panicum virgatum*)^59,60^. *Arabidopsis* GA signaling mutations led to an increase in the branch number^61,62^. These results indicated that the function of GA is to inhibit branching, contrary to its role in woody plants such as peaches. This finding suggested that GA may play a role in promoting branching in woody plants but may repress branching in herbaceous plants. These different effects may be due to the different degrees of plant lignification. Above all, *LfiGA2ox* may control the number of branches but does not promote the weeping trait in *Lagerstroemia*.

*LfiGRAS1* and *LfiGRAS2* belong to the GRAS family. GRAS family members such as *SCR* and *DELLA* are the main factors in GA signaling and regulation, thereby regulating various aspects of plant growth and development^49,50,57^. Decreasing *LfiGRAS2* transcription levels led to the malformation of new branches (Supplementary Fig. S10). When *LfiGRAS1* was transcribed at low levels, some new branches from the infected plants grew in a negatively geotropic manner. However, as the branches stretched, they gradually bent to elongate downward (Fig. 6). This effect may be due to factors such as gravity. When the downward bending moment caused by self-weight exceeds the upward bending moment caused by growth stress, the branches will grow in a weeping form. This phenomenon is highly similar to that observed in plants treated with GA3 (Fig. 2). However, neither of these treatments can significantly change the growth direction of existing branches.

The formation of tension wood on the side upper of the stem occurs to support the gravity of woody angiosperms^26^. When self-weight exceeds the tension, the branches weep. The initial stage of the development of tension wood and stem gravitropism are regulated by GA^63,64^. Moreover, the potential mechanism causing new shoots to grow upwards may be different from that in woody stems^65^. This difference may be the reason that GA3 treatment and decreased *LfiGRAS1* expression levels failed to rescue the woody stem phenotype. This finding suggests that GA may be associated with the early development of the weeping trait.

Paraffin sections showed that there were still no endodermal cells in the upright stems of *LfiGRAS1*-silenced plants (Supplementary Fig. S8). However, obvious phloem fibers were formed in the upright stems of *LfiGRAS1*-silenced plants. Subsequent experiments should further identify genes related to the promotion of endodermal cell differentiation from the perspective of endodermal cells to investigate the cause of the weeping trait in *Lagerstroemia*. The new upright stems produced in this study did not result from the restoration of their gravitropic responses through the production of endodermal cells. All directional growth in plants appears to involve competition between gravity and light. *PIFs* are responsible for integrating factors such as phytochromes, light, circadian clock, and GA signals^66^. DELLA proteins are considered to function as negative elements in GA signaling and integrate several members from the *PIF* family^67–69^. *PIFs*, which belong to the circadian rhythm-plant pathway, were significantly enriched in groups A, B, and C (Supplementary Fig. S4d, e, f), which implied that *PIFs* are associated with the weeping trait.

We expected that the phenotypes would be rescued due to the increase in the GA content, which changed the response to light in this study. We identified ten *LfiPIFs*, four of which were highly expressed in the weeping lines. All *LfiPIFs* were expressed at the highest levels in the buds (Supplementary Fig. S12). This result implied that PIFs may be associated with the early development of the weeping trait. Both GA3 treatment and the decreased expression levels of *LfiGRAS1* changed the growth direction of the new shoots but failed to rescue the phenotype of the woody stem. Similar to *LfiPIFs*, both GA3 and *LfiGRAS1* may play a role during early development. The upstream genes of *PIFs*, *phyA*, and *phyB*, were differentially expressed in the upright and weeping lines (Supplementary Fig. S12). Therefore, *LfiPIFs* and GA-related genes may act together to promote the weeping trait in crape myrtle.

Taken together, the results of this study indicate that weeping plants of *Lagerstroemia* lack endothelial cells. The direction of new branch growth can be changed by GA3 treatment. To further assess the reasons for the weeping trait, the axillary buds, axillary shoots, and stems from weeping and upright lines were examined via comparative transcriptome analysis. According to the transcriptome analysis and WGCNA, the expression levels of three genes involved in GA synthesis and signal transduction pathways were reduced in *Lagerstroemia* via the VIGS approach. The integration of the results suggested that GA is pivotal in the generation of weeping branches, and that GA promotes upward shoot orientations and narrow branch angles. Weeping architectures are desired for ornamental species. Our results provide an important foundation for improving branch orientations and breeding in *Lagerstroemia*. In addition, understanding the genetic basis of weeping may contribute to the identification of genes whose manipulation will benefit agricultural productivity.

## Supplementary information

Fig. S1 Material phenotype

Fig. S2 Analysis of transcriptome annotations

Fig. S3 Analysis of DEGs from the intersection of groups A, B and C

Fig. S4 KEGG functional analysis of DEGs in groups A, B and C

Fig. S5 Correlation analysis of the WGCNA modules and traits

Fig. S6 KEGG pathway functional enrichment of the four core modules

Fig. S7 Silence fragment construct maps of genes used in VIGS

Fig. S8 Paraffin section and growth phenotype of the LfiGRAS1-silenced plants

Fig. S9 Growth phenotype and expression analysis of the LfiGR2ox-silenced plants

Fig. S10 Growth phenotype and expression analysis of LfiGRAS2-silenced plants.

Fig. S11 Validating transcriptome data using qRT-PCR.

Fig. S12 Expression analysis of genes associated with light.

Table S1 Sampling schedule

Table S2 Primers used for qRT-PCR

Table S3 Primers used for the VIGS silencing fragment

Table S4: Annotations of all unigenes in the seven databases

Table S5 Transcript quality metrics

Table S6: Annotation summary

Table S7: Unigenes in four core modules

File S1 Sequence of all unigenes

File S2 Sequence of LfiGA2ox, LfiGRAS1 and LfiGRAS1

## Data Availability

The raw sequence data reported in this study have been deposited in the Genome Sequence Archive of the BIG Data Center (Beijing Institute of Genomics (BIG), Chinese Academy of Sciences) under accession number CRA001957 and CRA001957 is publicly accessible at https://bigd.big.ac.cn/gsa.
