# Supplementary figures and images for: Transcriptome profiles reveal that gibberellin-related genes regulate weeping traits in crape myrtle
Source: Hortic Res. 2020 Apr 1;7:54. doi: 10.1038/s41438-020-0279-3 (PMC7109059; doi:10.1038/s41438-020-0279-3)

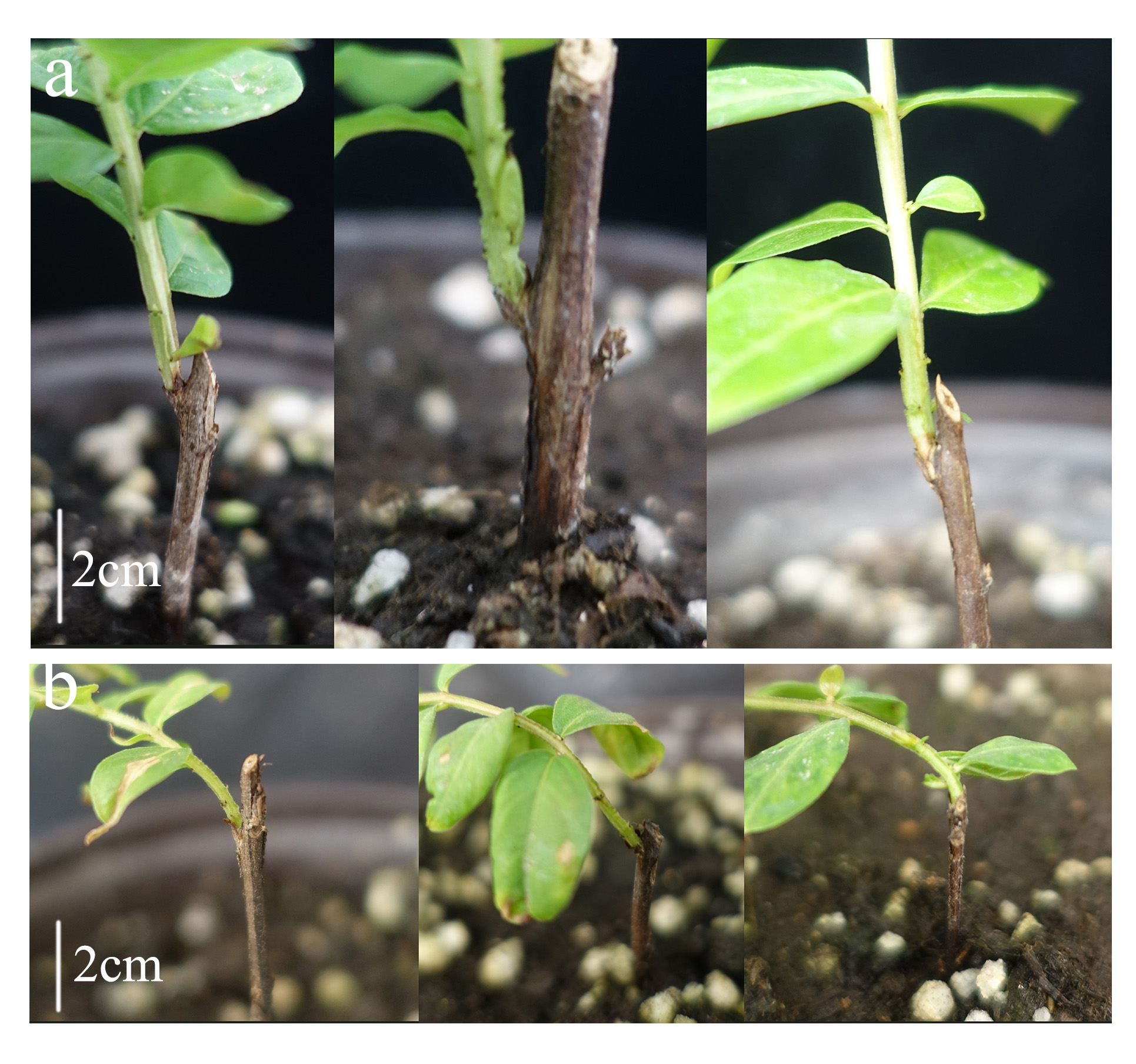

Supplement: Supplementary file 1 — Fig. S1 Material phenotype [file 41438_2020_279_MOESM1_ESM.jpg]

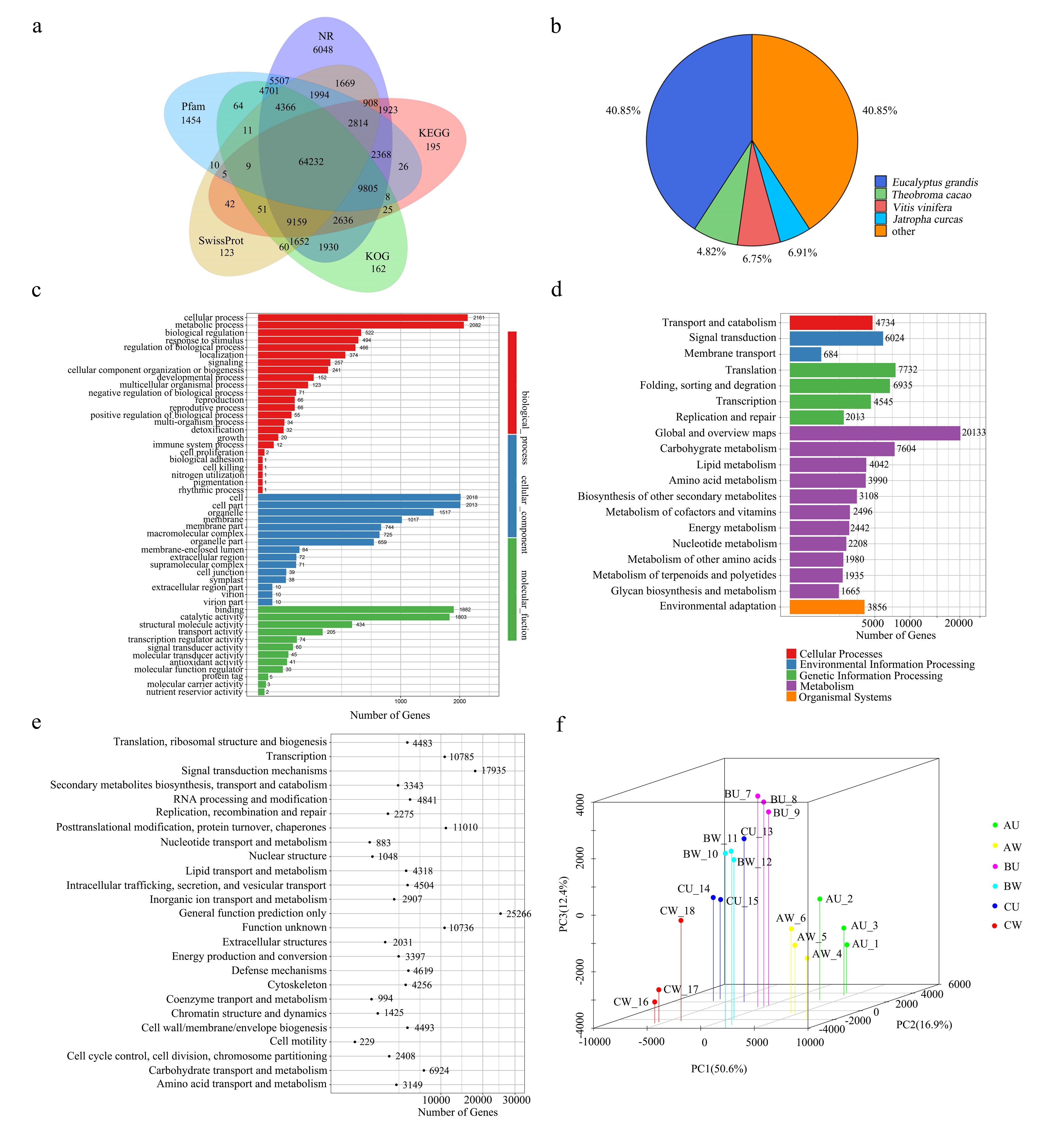

Supplement: Supplementary file 2 — Fig. S2 Analysis of transcriptome annotations [file 41438_2020_279_MOESM2_ESM.jpg]

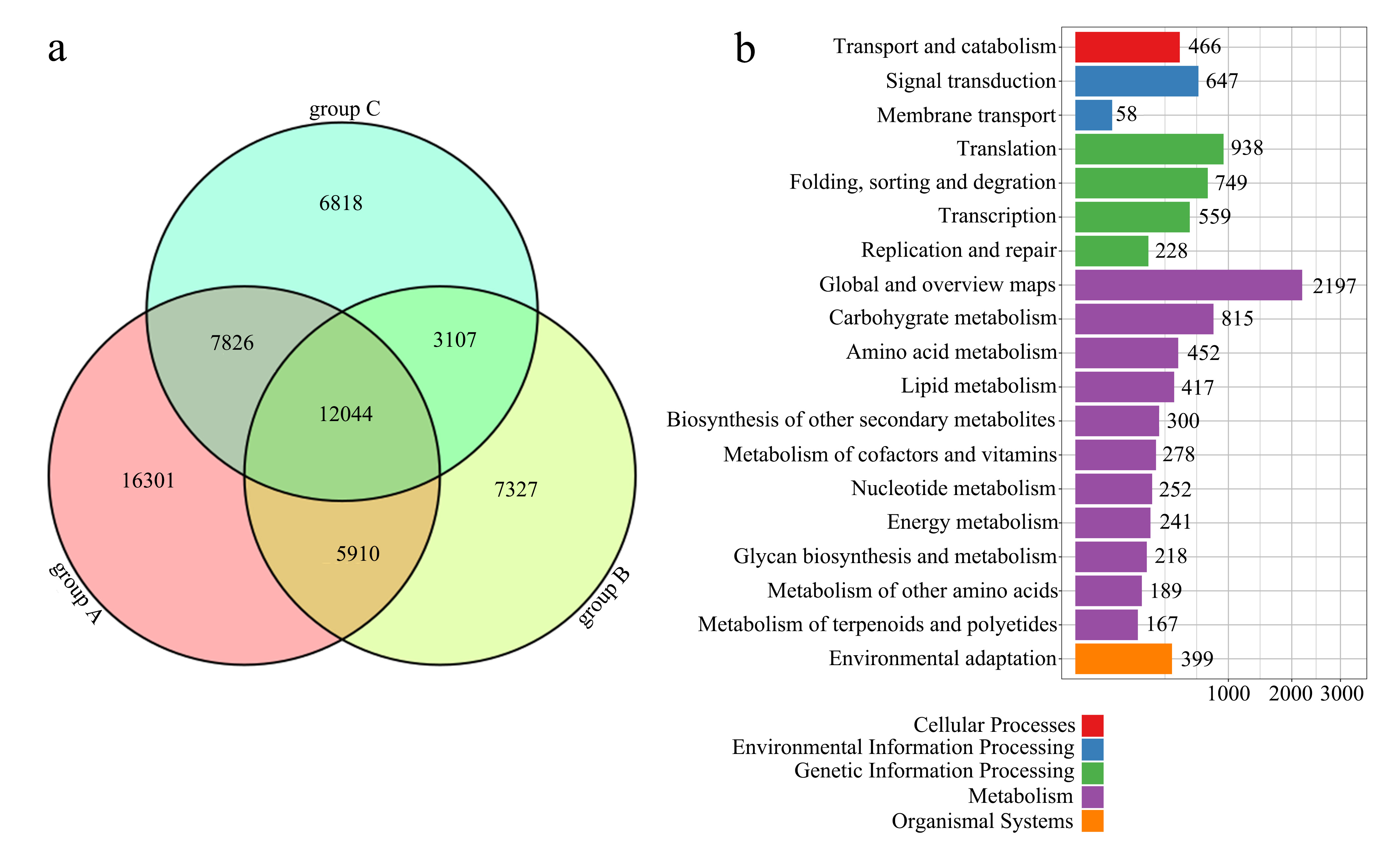

Supplement: Supplementary file 3 — Fig. S3 Analysis of DEGs from the intersection of groups A, B and C [file 41438_2020_279_MOESM3_ESM.jpg]

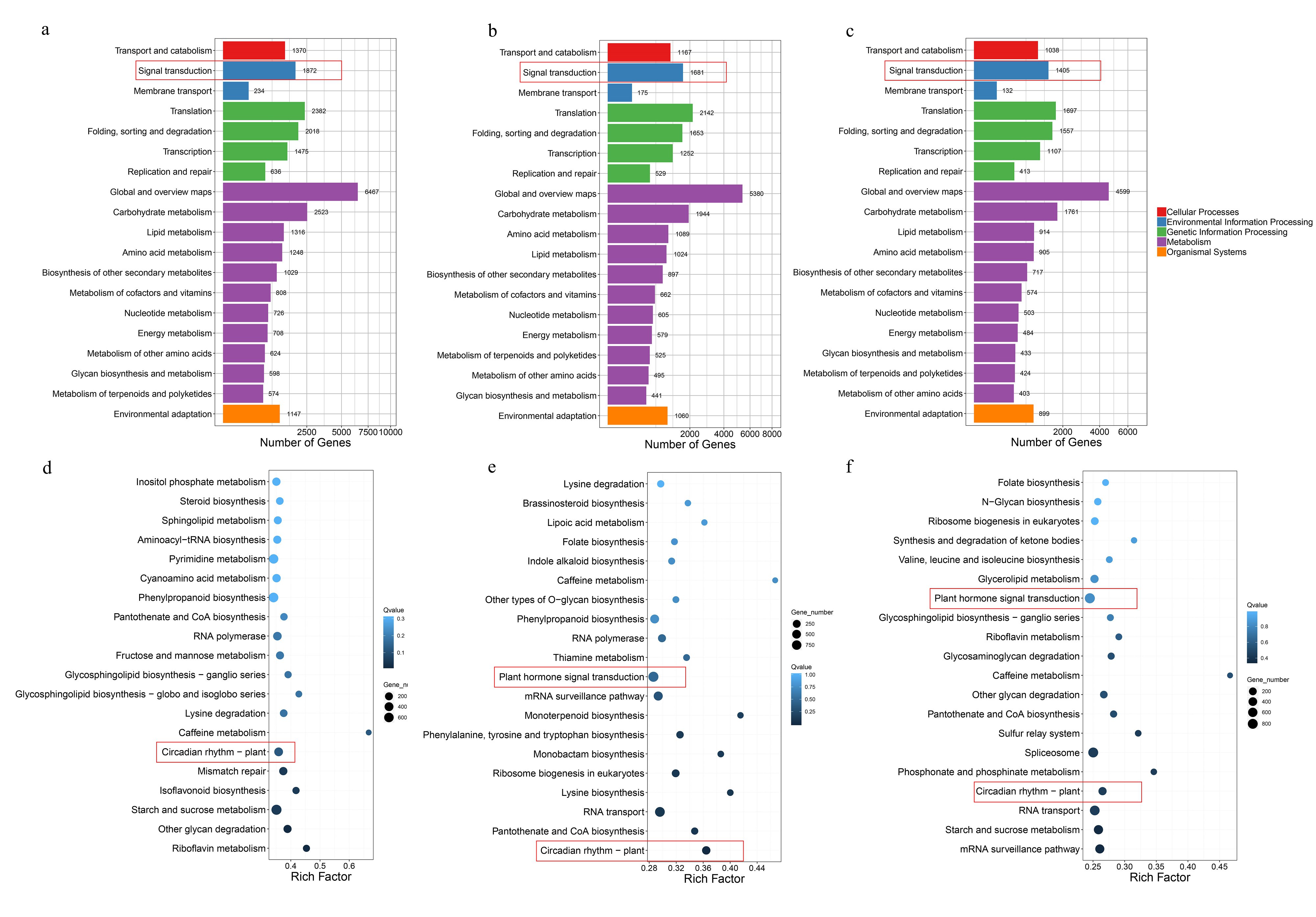

Supplement: Supplementary file 4 — Fig. S4 KEGG functional analysis of DEGs in groups A, B and C [file 41438_2020_279_MOESM4_ESM.jpg]

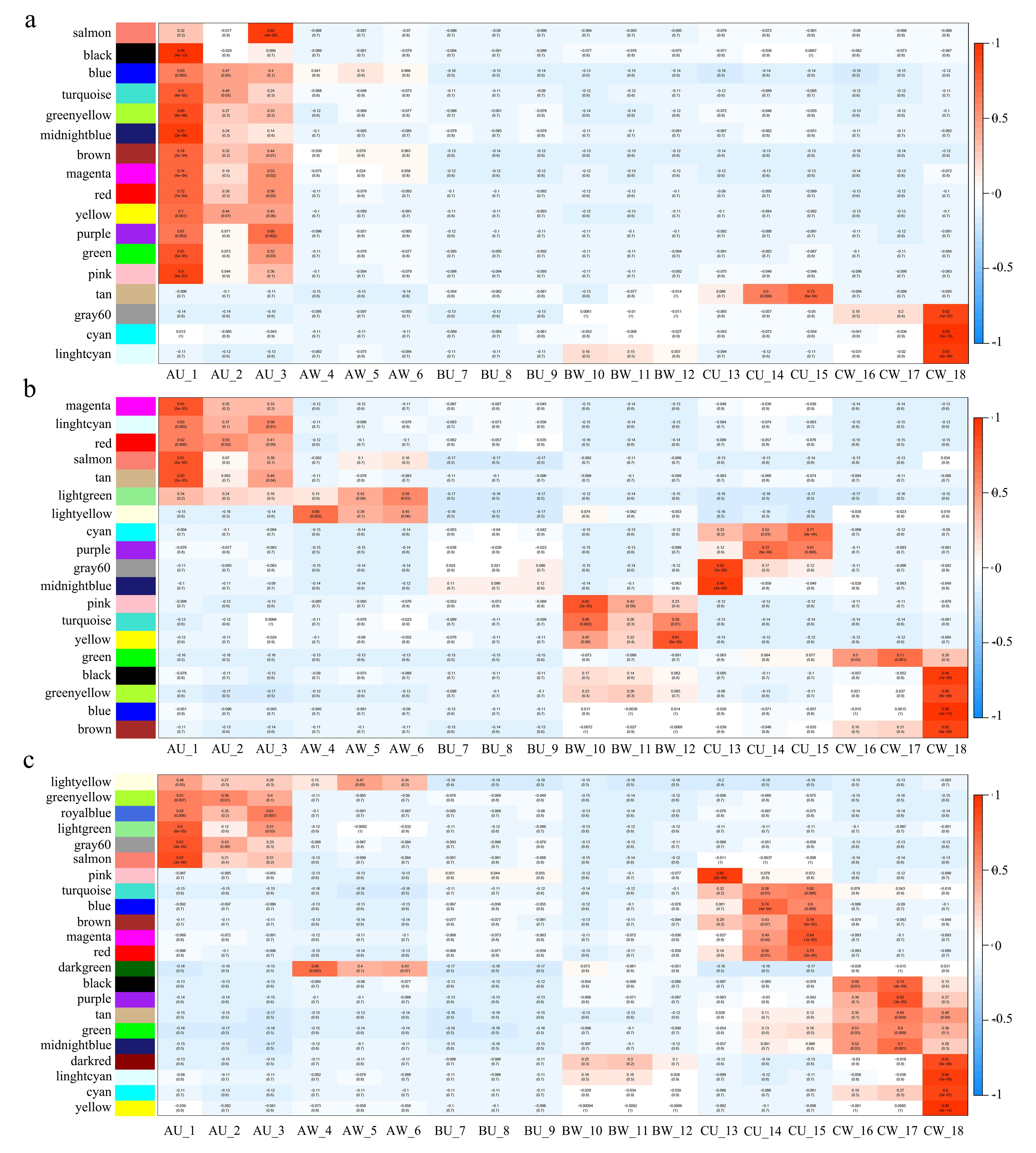

Supplement: Supplementary file 5 — Fig. S5 Correlation analysis of the WGCNA modules and traits [file 41438_2020_279_MOESM5_ESM.jpg]

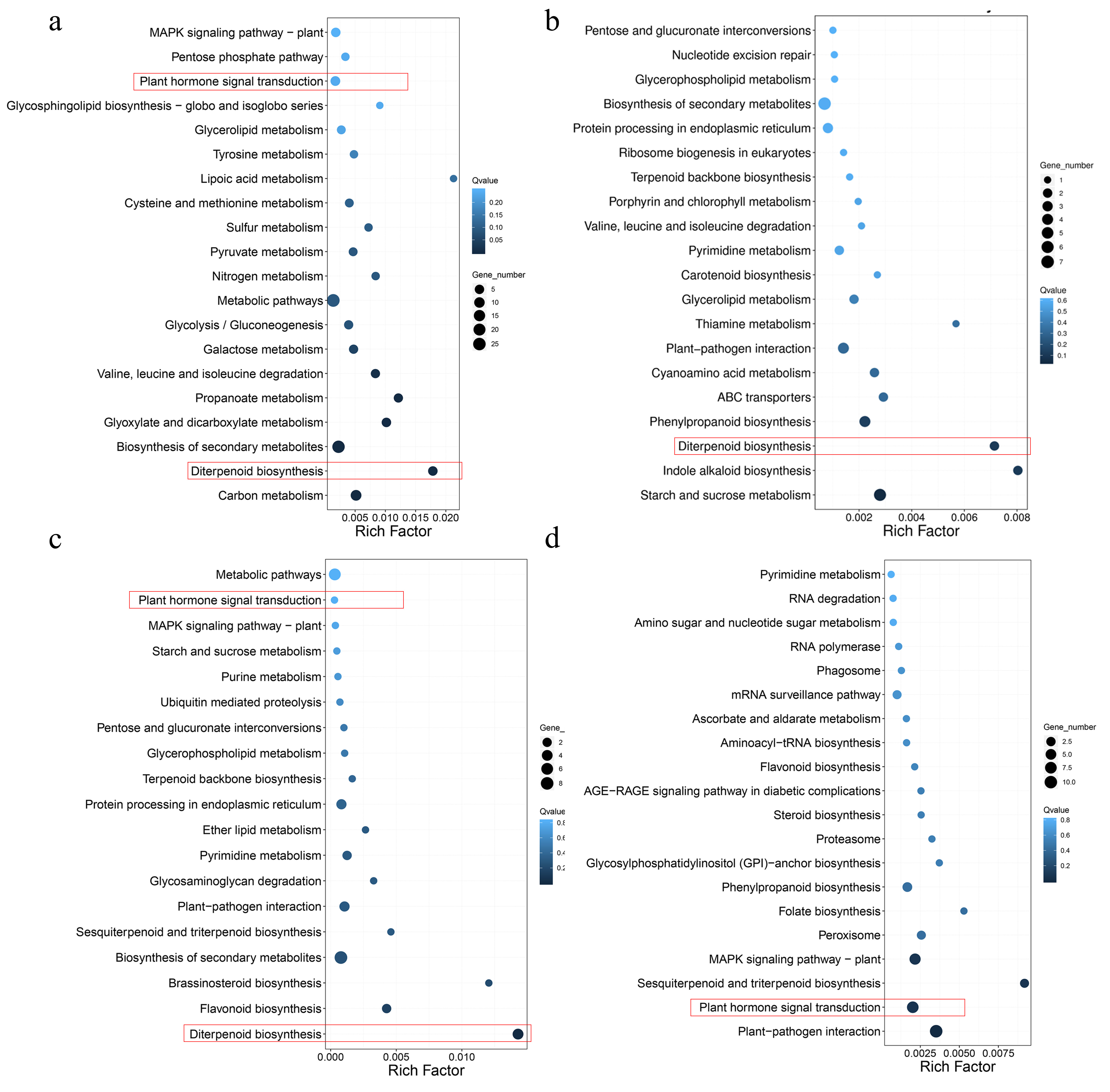

Supplement: Supplementary file 6 — Fig. S6 KEGG pathway functional enrichment of the four core modules [file 41438_2020_279_MOESM6_ESM.jpg]

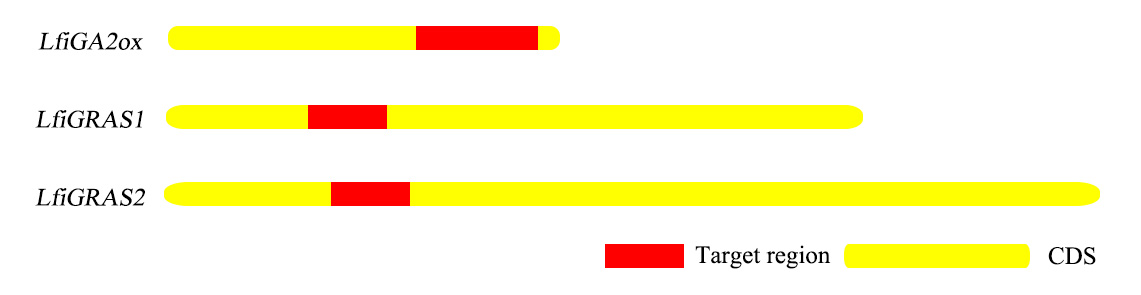

Supplement: Supplementary file 7 — Fig. S7 Silence fragment construct maps of genes used in VIGS [file 41438_2020_279_MOESM7_ESM.jpg]

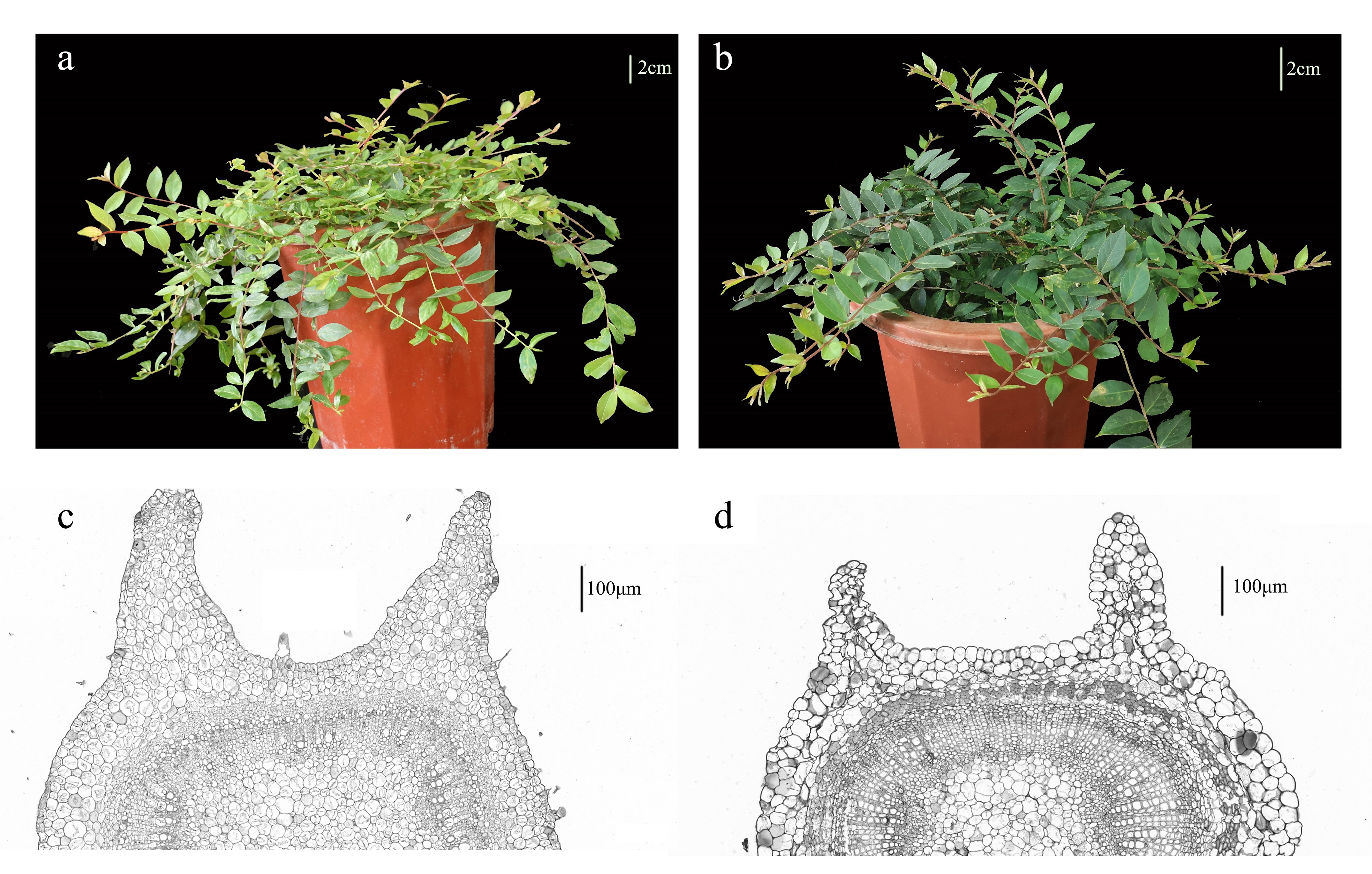

Supplement: Supplementary file 8 — Fig. S8 Paraffin section and growth phenotype of the LfiGRAS1-silenced plants [file 41438_2020_279_MOESM8_ESM.jpg]

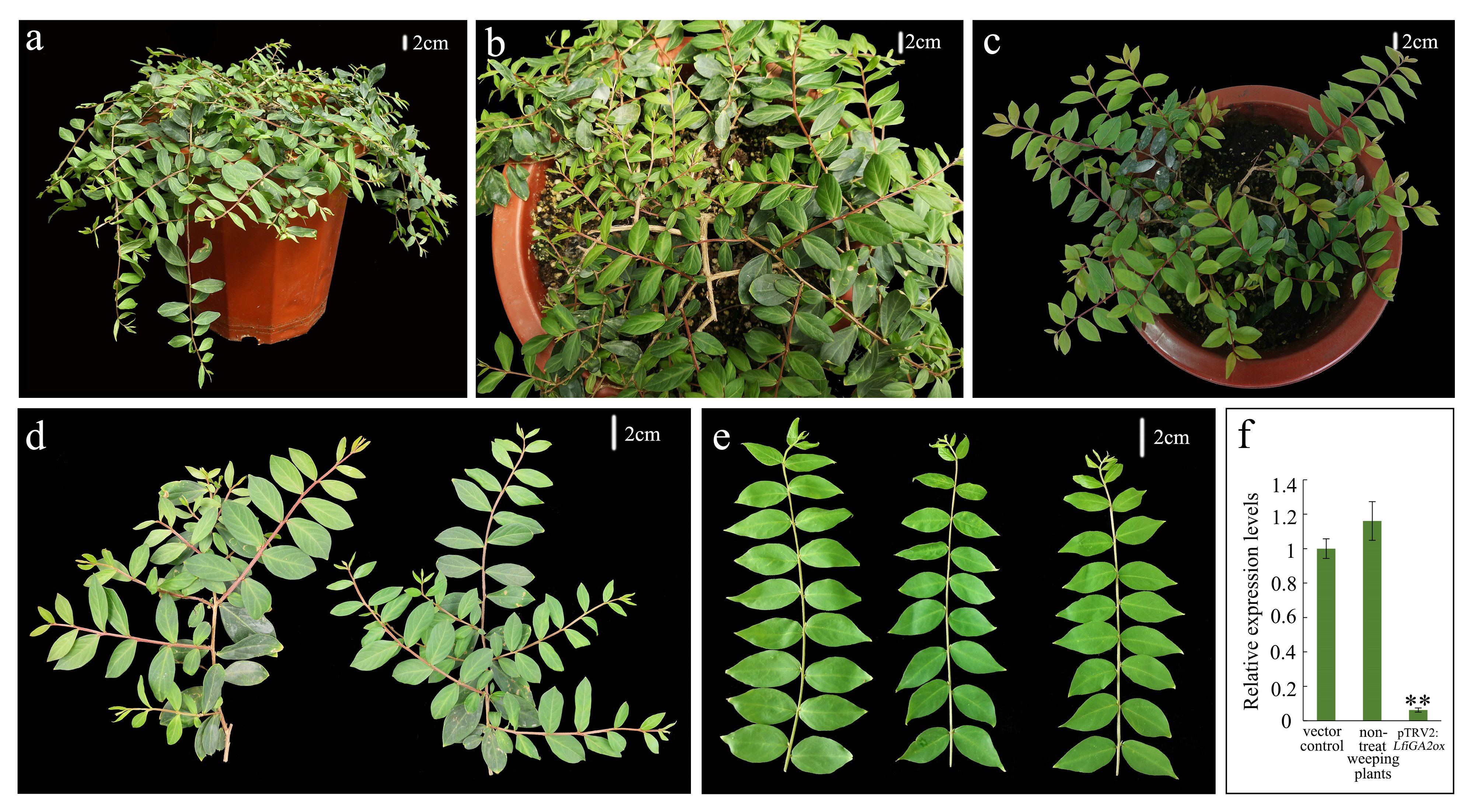

Supplement: Supplementary file 9 — Fig. S9 Growth phenotype and expression analysis of the LfiGR2ox-silenced plants [file 41438_2020_279_MOESM9_ESM.jpg]

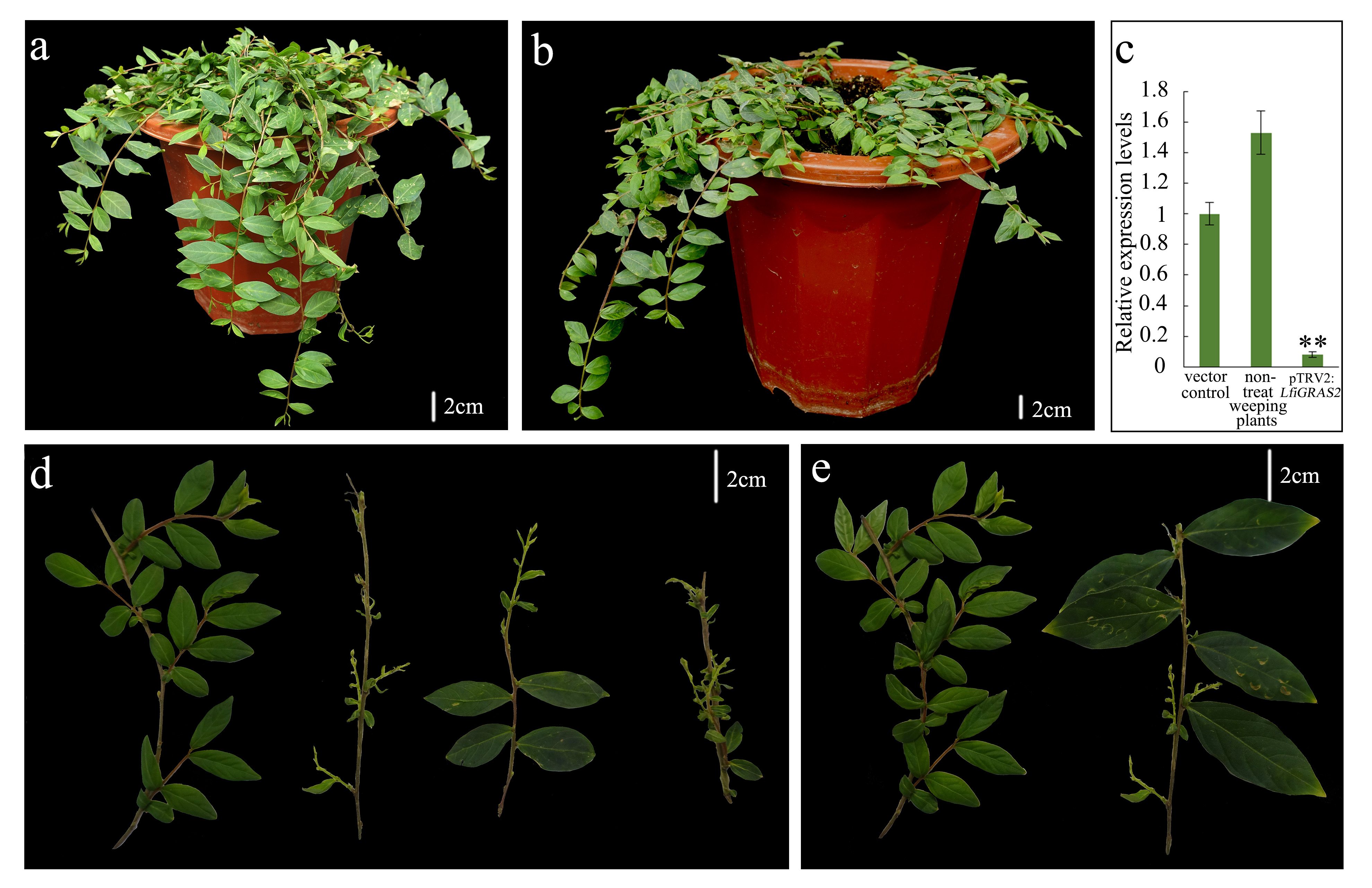

Supplement: Supplementary file 10 — Fig. S10 Growth phenotype and expression analysis of LfiGRAS2-silenced plants. [file 41438_2020_279_MOESM10_ESM.jpg]

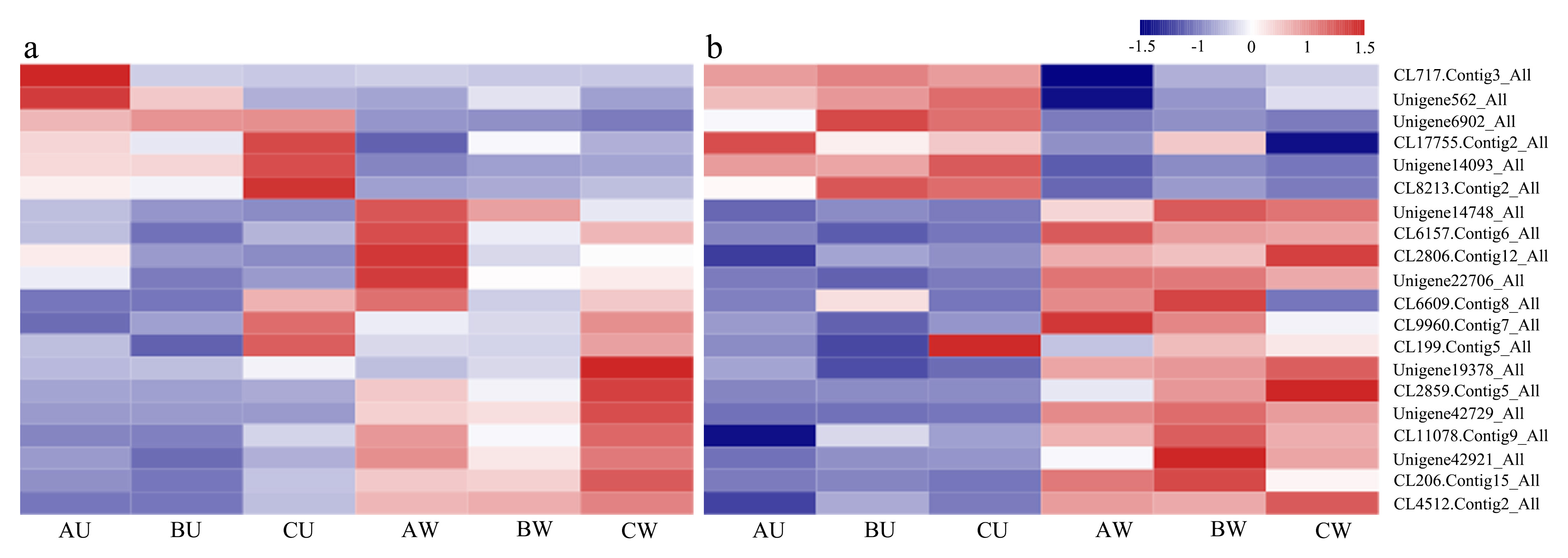

Supplement: Supplementary file 11 — Fig. S11 Validating transcriptome data using qRT-PCR. [file 41438_2020_279_MOESM11_ESM.jpg]

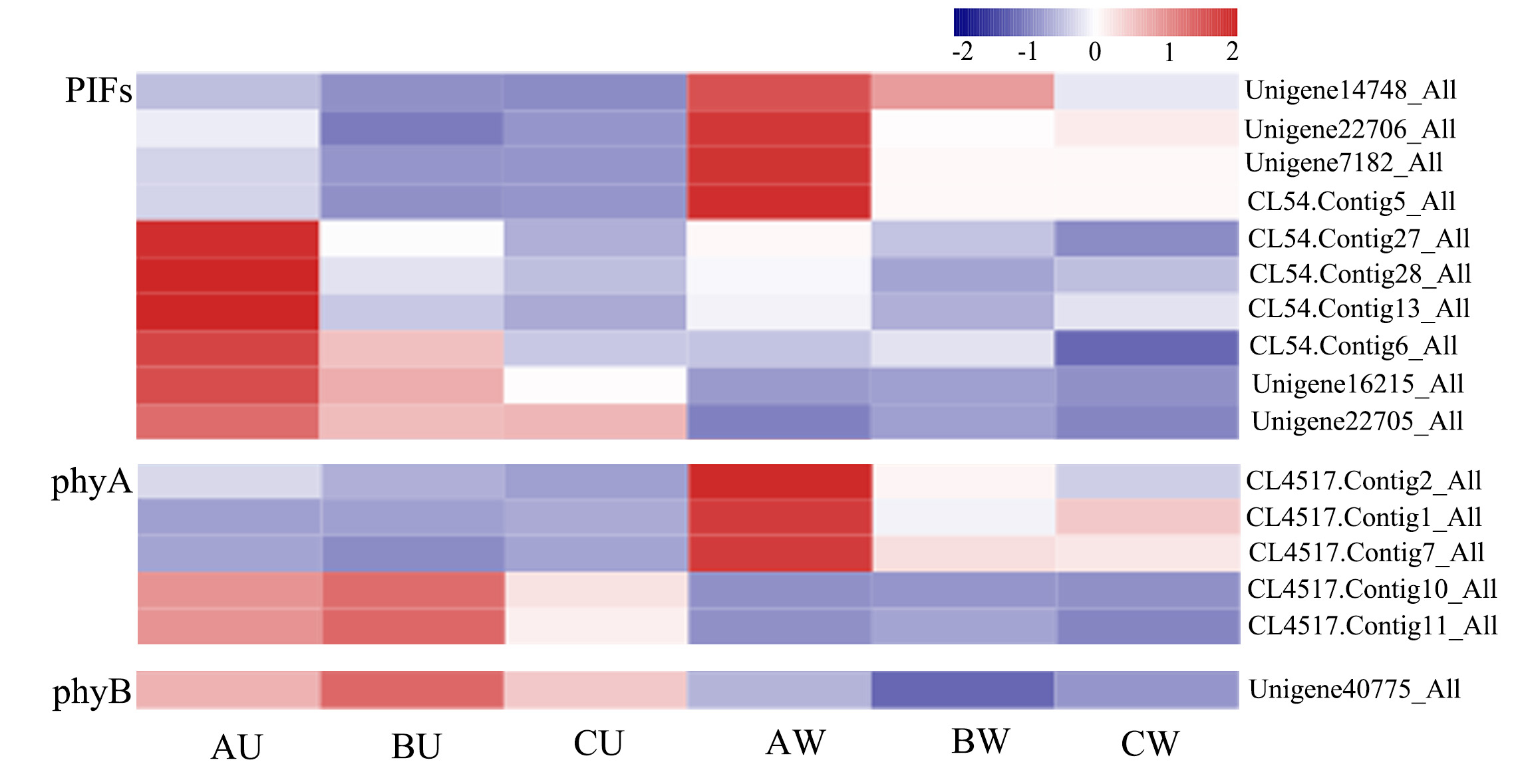

Supplement: Supplementary file 12 — Fig. S12 Expression analysis of genes associated with light. [file 41438_2020_279_MOESM12_ESM.jpg]
