# Supplementary material for: Transcriptome profiles reveal that gibberellin-related genes regulate weeping traits in crape myrtle
Source: Hortic Res. 2020 Apr 1;7:54. doi: 10.1038/s41438-020-0279-3 (PMC7109059; doi:10.1038/s41438-020-0279-3)
Supplement: Supplementary file 21 — File S2 Sequence of LfiGA2ox, LfiGRAS1 and LfiGRAS1 [file 41438_2020_279_MOESM21_ESM.docx]

>*LfiGA2ox*

ATGTTCTTACCCAAACCCCCTTTCTCATGTCTCTCCAATCGTGATTCTTCCGAGTCCGAG

TCCTTGCCCCTGATAGACCTCGCACAAACAGAGTCCAAGGACCTCATTGTGAAGGCCTGTGAGGACTTCGGGTTTTTCAAAGTCGTCAACCACGGGGTGCCCCTCGAGTTCATCTCAGCATTGGAATCTGAAGCAATAAAATTCTTCTCATTGCCCCTCTCCGAGAAGGAAAGGGCAGGGCCTCGACCTCCCAGTCCATTCGGCTATGGAAACAAGAATATCGGGCTGAATGGGGATGTTGGTTGGCTCGAGTACCTTCTCCTGACAACGAACCCCGATTTTGACTCCAGCAAGCTCCCATTAGTCTTCCGGGAAAACCCAGAAATATTCCGGGGCGCTTTGAATGATTACATAACAGCAATCAGGAACATGGGTTGCGAGATTCTGGAATTAATGGCGGATGGTTTGAAGATTCAACAGCGGAATGTGTTCAGCAAACTCTTAAAGGACCAACGGAGCGACTCTGTTTTCAGAATCAACCATTACCCTCCAAGGACGGAGCTTGACCGAGCCCTGAATGGAAGGGACGTGATCGGGTTCGGAGAGCACACAGATCCCCAGATAATTTCGATCCTGAGATCAAACGGGGTGTCGGGGCTGGAGATACAGAAGAGAGACGGGAGTTGGATTTGTGTTCCGCCGGACGAGAGGTCGTTCTTCGTCAATGTCGGCGACTCCCTACAGGTGATGACCAATGGAAGGTTCCGAAGCGTGAAGCACAGGGTGAAGACCAATGGAGAGAAGTCGAGGATTTCAATGGTGTACTTTGGGGGCCCGCCGCTCAGTGAGAGGATCGGTCCGCTGCCGTCCCTGGTGAGGGGAAACGAGAGCCTGTTGTACAGGGAATTCACTTGGTTCGACTACAAGAAGTCGGCCTACGCTACGAGGTTGTCGGATAATAGGCTCCAGCACTTTGAGCGTATAGCTGCATCCTGA

>*LfiGRAS1*

ATGAAGAAGAGGGATCACCAAGACAGCTGTAGCAGCTCCTATGGCGGCGACAGCGGCTCTTCTTCCTATGGTAAAGCCAAGGCTTGTTGGGGGGAGGAGGAGCAAGATCCCAGCGGCGGGGTGGACGAATTGCTCGCCGTCTTGGGGTACAAGGTCCGGCCTTCCGACATGGCGGATGTGGCCCTCAAGCTCGAGCAGCTCGAGATGGTCATGGGCCAGGACGACGGCGCCTCCTTGTCCCAACTCCAGAACGACACCGTTCACTACAACCCCTCCGACCTCTCCGCCTGGGTCCAGACTATGCTTTCCGAGCTCAACGCTCCTGTTCCCGACCCGGTTCAGTCCCTCCATACAAACCTCGACTTCTCCGGCCAGCTTAACCGAGTCACCGCAGAGTCGATTCAGTCGCCGTTCCCCTTCTCCGACGATTCCGAGTACGACCTCAGCGCTATACCCGGGGTCGCCGCCTACCCTCCTCCTTTGCCCGAGCCCGTCCGCAATAAGCGGATGAAGACAACGATTGGCTCCTCAAGCCCTAATATGAATCTTTCTTCGTCATCTTCCTCCTCTTCTGTGGTGGGTGCTCCGGAGTCAACCCGGCCGGTTGTGCTCGTTGACTCGCAGGAAACTGGTGTTCGGCTCGTTCACACACTTCTGGCTTGCGCGGAGGCGGTCGAGCAGGACAACCTGAAGCTCGCGGAGGCGCTGGTTAAGCACGTGGGTCTGCTCGCGGTATCGCAGGCCGGAGCCATGAGGAAGGTCGCCACCTGCTTCGCGGAGGCGCTCGCCCGCCGGATTTACAAGATCTACCCGCAGGACTCCCTGGACGCGACCTCCTACAACGACATCCTTCAGATGCACTTCTATGAGACTTGTCCGTACCTCAAGTTCGCCCACTTCACGGCCAACCAGGCCATCCTCGAGGCTGTCGGCGCGGCCAACCGGGTCCATGTGATTGACTTCAGCCTCAAGCAGGGGATGCAGTGGCCGGCCCTTATGCAGGCCCTTGCTCTTCGTCCCGGCGGCCCACCAGCATTCCGGCTCACTGGGATTGGTCCTCCTCAGCCCGACAACACCGACGCCCTTCAGCAGGTAGGGTGGAAGCTGGCCCAGCTGGCCGAGACGATTGGGGTCGAGTTCGAGTTCAGAGGTTATGTGGCCACGAGTCTGGCTGATATCGAGCCTGCCATGCTTGACCTTCGCCCGCCTGAGGTGGAGGCCGTAGCTGTCAACTCAGTCTTCGAGCTCCACCGCCTACTAGCACGCCCTGGCGCAATTGAGAAGGTTCTCAGCTCTATCAAGGCCATGAAGCCCAAGATTGTGACCATAGTTGAGCAAGAGGCGAACCACAACGGCCCAGTCTTCCTGGACCGGTTCACCGAGGCCCTGCACTACTACTCGAGCCTATTTGACTCACTGGAAGGGTCTGGCCTGACCCCGGAGAGTCAGGACCTGGTGATGTCGGAGGTCTACCTTGGGAGCCAGATTTGCAACGTAGTAGCCTGTGAGGGGCCAGATCGGTTAGAGAGGCACGAGACGCTGGCCCAGTGGCAGTCTAGGCTGGGCTCGTCTGGCTTCGACCCGGTTCATCTCGGATCGAATGCATTCAAGCAGGCAAGCATGCTGCTGGCCCTGTTTGCTGGCGGGGACGGGTATCGCGTAGAGGCATACAATGGGTGCCTCATGCTTGGGTGGCATACTCGGCCGCTCATCGCCACATCAGCTTGGCAGCTTGCCCCGACGGTCACGAGAAAGTCTCGTATGTAA

>*LfiGRAS2*

ATGGCTGCCGCCTGTGCACTGCTAAGCTCCGACGCTGATAATGCTGCCTCCCCCAACGGACACAGCCGCCGCGACTCCCCCTCTCTCATGAGCTCCGCCGGCAGCAACCTCCACCACCACACGGACCCCCTCCTCCTCCTCAGCGCCGCCGCGCCTTCTCCTCCTGCCTTTCCCCGTCAGCAGCAACACTGCAACCTCAGCAGCAACAGAATGGTGAGGAAGCGGGTAGCCTCCGAGATGGAGGCCCTCGCTGCCGGTTGCGCCGGATCCGATTCCTATTTCCGGCTCCCCCGTCGGGCCGGGACAAATCCTAACGGCCTCGACGGTGATTCGGCGCAGTCGTTTCAGAGTCATCCTCCCACCGCCATTACTTTCCCCAACCACTCCACGAGCGCTGCTACACCGAGTCTGAACGTGACATCACCCTGCTCGTTTTCATCCCCAGCGGTCATGACCACCAACGGCACTCCTGCAACCGCCTCTCTCCCGTACATCGACACCTCCCTCCCTCCTCCCGCAAATCATCCGACGTCGTCTGCCGCCGTTGAGGCGCCGGCTCTCTGCGTCTTCTCCGGGCTGCCTCTGTTTCCCCCGGAGAGAAACCGCTCCTCCGCTCCCCTAACGGCACCTGCTGCGTCCTCTGCCGGTAACATGGACGACGGCTCTGCGGCGACGTGGGTCGATGGCATCATAAAGGACCTCATACACGGCGCCGCCAACGTGTCCATCCCTCAGCTCATACAGAACGTCCGGGAAATCATCTACCCCTGCAACCCTAGCCTCGCCGCCGTCCTCGAGCTCAGGCTACGCTCCCTAATAGAGCCGATGGCTTCCTCCGCGTCCATTCTTCCCCCGCAGATGCCGTTCAACCAGCTCGGCAGTTCTAGCCTCCGCCTTAACCTCGAATCCGCTGCTCTCGACAGCAACGCTTACCCCTCGGAACCCGCCTCTTACTTGAACTACGCGGTACCGCTGCCGTCGAGCGCCCCTGCGGTCCACCGTCTCCACGAGCACCAGGCTATTCAACCGGAGGCCCAGCCAGAGCCGCTGCCCCGGCAGCACCAGCAACATCCGCAACCGCCGGTGTCGGAGCCGGAGAAGTCGTCCCCCTCCGCTGATGTGGCATCATCGGCAGACAACGTAACAGGTGCTTCCTCCATTGCCGCCGCAGCAGCCGCAGCGGCGACCGCTCTGGCGAGGGAAAAGGAAGAGATGAAGCAGCGGAAGAAGGACGAGGAGGGGCTTCACCTGCTGACTTTGCTCCTTCAGTGCGCGGAGGCGGTCTCGGCAGACAACCTCGAGGAGGCTAACAGAATGCTCATCGAGATCTCTGAGCTGTCGACGCCCTATGGGACCTCGGCTCAGCGGGTGGCAGCCTACTTCTCGGAGGCCATTTCCGCCCGCCTCGTGAACTCTTGCCTGGGGATCTACGCCTCCCTCCCGGCGGCCCCGCACAGCTACAAGATCTCCTCTGCCTTCCAAGTATTCAATGGCATCAGCCCCCTCGTGAAGTTCTCCCACTTCACGGCGAACCAAGCCATTCAAGAGGCCTTTGAGAGAGAGGACAGGGTCCACATCATCGACCTCGACATAATGCAGGGCCTCCAGTGGCCGGGGCTCTTCCACATCCTGGCCTCCCGTCCCGGCGGCCCGCCGCACGTGAAGCTGACGGGGCTGGGGAACTCCATGGAGGCGCTGGAGGCCACGGGGAAGCGGCTCGCTGAGTTCGCCGACAAGCTGGGCTTGCCCTTTGAGTTCTGCCCGGTGGGGGACAAGGTTGGGAACTTAGATCCTGAGAGGCTCAACGTCAGTAAGAGGGAGGCCGTGGCCGTCCACTGGCTCCAGCACTCCCTCTACGACGTCACCGGCTCCGACGCCAAGACGCTGTGGCTCCTGCAGAGGTTGACACCGAAGGTGGTGACAGTGGTGGAGCAGGACCTGAGCCAGGCTGGATCGTTCCTAGGTCGGTTTGTGGAGGCCATCCACTACTACTCGGCCCTGTTCGATTCACTCGGGGCAAGCTACGGAGAGGAGAGTGAGGAGCGGCACGTGGTGGAGCAGCAGCTGTTGTCTCGCGAGATACGCAACGTGCTCGCCGTGGGTGGGCCCTCTAGGAGTGCTGCCGCTGCTGAGAGGCCCAGCGGCAGCTGGAGAGACAAGTTCCGGCAGGCCGGATTCAGGTCTGTTTCACTCTCCGGCAACCCCGCTAACCAGGCCACCCTCCTACTTGGGATGTTCTCTTGTGATGGGTACACCCTGGTTGAGGACAATGGGACACTCAAGCTCGGGTGGAAGGACCTGTGCCTCCTCACCGCTTCCGCTTGGAGCCCCGTCATCTCCTCTTCGCCGAATCTGCACGTGAACCCAAACCCGACTGGGCATTTTTCTCGTTGA
